# Eco-Friendly Iron-Humic Nanofertilizers Synthesis for the Prevention of Iron Chlorosis in Soybean (*Glycine max*) Grown in Calcareous Soil

**DOI:** 10.3389/fpls.2019.00413

**Published:** 2019-04-05

**Authors:** María T. Cieschi, Alexander Yu Polyakov, Vasily A. Lebedev, Dmitry S. Volkov, Denis A. Pankratov, Alexey A. Veligzhanin, Irina V. Perminova, Juan J. Lucena

**Affiliations:** ^1^Department of Agricultural Chemistry and Food Science, Autonomous University of Madrid, Madrid, Spain; ^2^Kurnakov Institute of General and Inorganic Chemistry, Russian Academy of Sciences, Moscow, Russia; ^3^Department of Materials Science, Lomonosov Moscow State University, Moscow, Russia; ^4^Department of Chemistry, Lomonosov Moscow State University, Moscow, Russia; ^5^Department of Chemistry and Physical Chemistry of Soils, V.V. Dokuchaev Soil Science Institute, Moscow, Russia; ^6^National Research Center “Kurchatov Institute”, Moscow, Russia

**Keywords:** iron nanoparticles, iron nutrition, humic substances, leonardite, ^57^Fe, soybean, ferrihydrite

## Abstract

Iron deficiency is a frequent problem for many crops, particularly in calcareous soils and iron humates are commonly applied in the Mediterranean basin in spite of their lesser efficiency than iron synthetic chelates. Development and application of new fertilizers using nanotechnology are one of the potentially effective options of enhancing the iron humates, according to the sustainable agriculture. Particle size, pH, and kinetics constrain the iron humate efficiency. Thus, it is relevant to understand the iron humate mechanism in the plant–soil system linking their particle size, characterization and iron distribution in plant and soil using ^57^Fe as a tracer tool. Three hybrid nanomaterials (F, S, and M) were synthesized as iron-humic nanofertilizers (^57^Fe-NFs) from leonardite potassium humate and ^57^Fe used in the form of ^57^Fe(NO_3_)_3_ or ^57^Fe_2_(SO_4_)_3_. They were characterized using Mössbauer spectroscopy, X-ray diffraction (XRD), extended X-ray absorption fine structure spectroscopy (EXAFS), transmission electron microscopy (TEM) and tested for iron availability in a calcareous soil pot experiment carried out under growth chamber conditions. Three doses (35, 75, and 150 μmol pot^-1^) of each iron-humic material were applied to soybean iron deficient plants and their iron nutrition contributions were compared to ^57^FeEDDHA and leonardite potassium humate as control treatments. Ferrihydrite was detected as the main structure of all three ^57^Fe-NFs and the plants tested with iron-humic compounds exhibited continuous long-term statistically reproducible iron uptake and showed high shoot fresh weight. Moreover, the ^57^Fe from the humic nanofertilizers remained available in soil and was detected in soybean pods. The Fe-NFs offers a natural, low cost and environmental option to the traditional iron fertilization in calcareous soils.

## Introduction

Iron (Fe) is an essential micronutrient for humans and plants. Iron deficiency is very common in the human diet and affects an estimated two billion people in the world (Briat et al., 2015). Iron chlorosis is a widespread agricultural problem occurring in about 30–50% of cultivated soils (Cakmak, 2002) and one of the major limiting factor of crop production in calcareous soils. Farmers apply iron synthetic chelates to alleviate iron deficiency in cash crops. Despite the high costs of these fertilizers, they tend to lixiviate and the chelating agents may avoid the precipitation and enhance mobilization of heavy metals (Ylivainio, 2010). Many crops are sensitive to the iron chlorosis, such as citrus and fruit trees but soybean (*Glycine max* L.) is one of the most studied iron Strategy I plant (Fuentes et al., 2018). Moreover, soybean production reaches levels of about 230 million metric tons per year across the world (Vasconcelos and Grusak, 2014) and this legume is a highly nutritious crop which contains more protein (40%) and oil (20%) than any other ordinary food source (Bolon et al., 2010).

According to the United Nations [UN] (2013), the rapidly growing world population is projected to reach 9.6 billion by the year 2050 and Food and Agriculture Organization of the United Nations [FAO] (2017) has predicted that the global grain production is required to increase by 70% to meet these demands. Therefore, new approaches should be developed for alleviation of iron deficiency in plants and new ecofriendly fertilizers are needed in order to enhance crop environmental quality. Iron fertilizers based on HSs extracted from lignites, such as leonardite, are used in the Mediterranean area (as liquid concentrates) in drip irrigation (Kovács et al., 2013). This kind of iron fertilizers is more ecofriendly than synthetic iron chelates but they are less efficient in correcting iron chlorosis. Moreover, field experiments have demonstrated that the synthetic chelate has a fast effect while the iron humate fertilizers provide increasing iron availability in the root–soil interface resulting in slow uptake of Fe by the plants (Cieschi et al., 2017). Kulikova et al. (2017) have demonstrated that only iron from very small and amorphous nanoparticles of ferric polymers incorporated into humic matrix is readily taken up by plants. Therefore, the synthesis of iron humates should be optimized for developing efficient NFs.

According to Naderi and Danesh-Shahraki (2013), NFs are the most important products of nanotechnology with regard to agriculture. Nanosized active ingredients (from 1 to 100 nm in diameter) have a large specific surface area that can result in significantly enhanced reactivity, and this feature increases absorption of nutritional elements and essential compounds for plant growth and plant metabolism (Janmohammadi et al., 2016). Many attempts has been made to prepare inorganic Fe nanofertilizers. As example Sánchez-Alcalá et al. (2012) synthesized nanosiderite (FeCO_3_) and demonstrated that it was highly effective in preventing iron chlorosis in chickpea and had a great residual effect. Ghafariyan et al. (2013) reported that low concentrations of superparamagnetic Fe-NPs significantly increased the chlorophyll contents in sub-apical leaves of soybeans in a greenhouse test under hydroponic conditions, suggesting that soybean could use this type of Fe-NPs as source of Fe and reduce chlorotic symptoms of Fe deficiency. However, the research on natural Fe nano-humate complexes is now in progress. Dholakia (2016) developed the preparation of nanoparticulate liquid organic fertilizers employing humic acids. In addition, Kulikova et al. (2017) have synthesized well-defined iron (hydr)oxide NPs of feroxyhyte stabilized by traces of HS (a model of iron-based engineered NPs) and water-soluble Fe-HS complexes of the proven high availability to plants tested their iron materials in wheat plants under hydroponic conditions. These promising results motivated us to follow the research on Fe NFs stabilized with humates.

According to Dimkpa and Bindraban (2017), up to now, the bulk of research in plant nanoscience either consists of experiments conducted in artificial media, such as nutrient solutions, agar, sand, or other non-soil media. Moreover, Liu and Lal (2015) recommend that micronutrient research should focus on enhancing the bioavailability (plant-uptake rate) of NFs to address the field leaching associated with the conventional micronutrient fertilizers and compare the beneficial effects of these micronutrient NFs with commercially available micronutrient counterparts [e.g., FeNPs vs. FeCl_3_ or Fe(EDTA) as Fe sources] under the field condition. Therefore, it is of particular importance to test the ^57^Fe-NFs in a soil system in a long-term experiment which would enable for completion of the full growth cycle crop in order to be closer to agronomical conditions. Since the efficacy of an iron fertilizer is related to the iron that the plants can take from the fertilizer, the use of iron isotopes is highly beneficial for monitoring iron uptake by plants (Cesco et al., 2002; Nikolic et al., 2003; Tomasi et al., 2013). The use of stable Fe isotopes instead of radioactive ones gives a high flexibility in the experimental designs and can include field studies, because special safety measurements and trained staff are not required. Moreover, long-term assays can be carried out without taking care of radioactivity decay over time. In addition, the generation of radioactive wastes is avoided (Benedicto et al., 2011). Many studies about ^57^Fe application in soils experiments (Nadal et al., 2012; Martín-Fernández et al., 2017a,b) were reported, but this work is the first one in preparing ^57^Fe-NFs and applying them in a calcareous soil.

Here, three ^57^Fe-labeled humic nanomaterials (F, S, and M) were synthesized using potassium humate as a parent humic material and ^57^Fe in the form of ^57^Fe(NO_3_)_3_ (product F) and ^57^Fe_2_(SO_4_)_3_ (products S and M), characterized for iron speciation and phase composition of nanoparticles, and tested for bioavailability to soybean iron deficient plants grown in calcareous soils under growth chamber conditions. This was to establish a link between the Fe-NPs characteristics and their behavior in the soil–plant system using ^57^Fe as a tracer tool.

## Materials and Methods

### Reagents

All reagents used were of recognized analytical grade, and solutions were prepared with type-I grade water (ISO 3696:1987, 1987) free of organic contaminants (Millipore, Milford, CT, United States).

### Synthesis of ^57^Fe-NFs

Prior to the synthesis, a known weight of leonardite potassium humate (C 34.9%, H 3.89%, N 0.72%, S 0.06%, Fe 0.45%) (Powhumus, Humintech Ltd., Germany) was dissolved in distilled water and centrifuged at 10,000 min^-1^ for 10 min to separate and discard any insoluble mineral components. The obtained solution contained 70 g L^-1^ of leonardite potassium humate (L solution) and was used for the further NF synthesis.

A ^57^Fe_2_(SO_4_)_3_ solution (0.20 M in ^57^Fe) was prepared from metallic ^57^Fe (Isoflex, 96.28% ^57^Fe isotopic enrichment) by dissolving 0.4008 g in 34 mL 1M H_2_SO_4_ and heating till complete dissolution. After that, two products (S and M) were obtained by interaction of potassium humate with ^57^Fe_2_(SO_4_)_3_ solution. In brief, the product S was synthesized as in Sorkina et al. (2014), 17 mL of 0.2M ^57^Fe_2_(SO_4_)_3_ solution was added dropwise to 14.3 mL of the L solution and pH was maintained at a value of 10 by adding slowly 1M KOH when needed. For the synthesis of the product M, 17 mL of ^57^Fe_2_(SO_4_)_3_ solution was slowly added to 40 mL of L solution, maintaining the pH at 9 with 1M KOH. The product M was prepared with the 90% of its maximum complexing capacity (Fe-MCC). Determination of Fe-MCC was conducted as described in Villén et al. (2007) and presented in the Supplementary Figure SM1. According to the obtained titration curve, an amount of 190 mg of Fe (III) per g org C^-1^ was necessary to obtain 200 mg of complexed Fe (III) per g org C^-1^ at the MCC.

Similarly, for the preparation of F product, a ^57^Fe(NO_3_)_3_ solution was prepared from the same metallic ^57^Fe by dissolving 0.2004 g in 5 mL HNO_3_ (70%, 1,401 g/mL density) and then diluted. The obtained solution was added dropwise to 32 mL of the L solution, maintaining the pH at 9 with 1M KOH.

In all syntheses described above, the final reaction mixtures were frozen “as is” using liquid N_2_ and freeze-dried using Labconco FreeZone freeze dry system (-50°C, 0.03 mbar pressure).

It should be noted, that the high hydrolysis rate is required to obtain ultradispersed (nanosized) iron (hydr)oxide nanoparticles from iron (III) sulfate or nitrate solutions. For this, we added ^57^Fe_2_(SO_4_)_3_ and ^57^Fe(NO_3_)_3_ dropwise but rapidly to the strongly alkaline medium of potassium humate solution and prevented the pH drop by simultaneous addition of KOH. The pH values between 9 and 10 were chosen to ensure formation of the disordered and chemically labile iron oxy-hydroxide phases instead of well-crystalline iron oxides like Fe_3_O_4_, Fe_2_O_3_ or rigid α-FeOOH.

The content of soluble iron in the synthesized fertilizers was determined using ICP AES. It was (in % mass) 2.9, 2.3, and 2.1 in the samples F, S, and M, respectively. The Fe:org C ratios were 0.27, 0.52, and 0.12 (g Fe g C org^-1^) in the samples F, S, and M, respectively.

### Characterization of ^57^Fe-NFs

The freeze dried preparations of ^57^Fe-NFs were exhaustively characterized using XRD, TEM with ED, EELS and energy filtered transmission electron microscopy (EFTEM), X-ray absorption spectroscopy (XANES and EXAFS) and Mössbauer spectroscopy. The XRD patterns were collected at CuKα on Rigaku D-MAX 2500 diffractometer in Theta/2Theta geometry. Reference samples, such as ferrihydrite and goethite were synthesized according to the procedure proposed by Schwertmann and Cornell (1992) and described by López-Rayo et al. (2015).

The TEM data were obtained with the use of Zeiss Libra 200MC microscope, equipped with monochromator and Omega-filter. For the TEM measurements, samples were dissolved in distilled water, dropped on the lacey-carbon coated copper grid for the few minutes with the following removal of the solution excess to reduce the concentration of mineral salts in the sample. Details of energy filtered TEM and SAED acquisition and processing are described in the corresponding part and in Supplementary Materials. Image processing was performed with the use of Gwyddion (Nečas and Klapetek, 2012), further data treatment – with the use of *scipy* and *matplotlib* (Hunter, 2007). The EELS spectra were acquired using the omega-filter, and integrated with DigitalMicrograph2 (DM2) software, Gatan. The variable slit of 3.5 eV width was placed in the Omega-filter to select the elastic part of scattered electrons. Two EFTEM images were collected with the background signal differing in intensity in the pre-edge energy area, and one image – with the combined signals of iron and background – was collected on the Fe M-line. Final iron distribution map was calculated using the DM2 software.

X-ray absorption spectra were measured at the STM beamline of Kurchatov Synchrotron Source facility, National Research Center “Kurchatov Institute”, Moscow, Russia. The Si (111) channel-cut monochromator was used. Ionization chambers with length of 10 cm filled with argon were used as detectors of incidence and transmitted beams. The samples were used as dry powders mounted onto the kapton tape. Thickness of each sample was adjusted to yield the absorption value of 3. All spectra for the iron-containing NFs under study and the iron (hydr)oxide references were measured by absorption using Fe foil as a reference sample. XAS of the parent humate (L) was measured using fluorescence due to the low iron content in this sample. The Amptek X-123 SDD detector was used for this purpose. Six spectra were acquired for each sample and averaged. All spectra were handled with the use of Athena software. The further modeling and refinements were done using the Artemis software. Refinements were performed by *k*^2^-weighted spectra in the range of 3–14 Å^-1^ in the *k*-space, and of 1–3.5 Å in the R-space, Hanning window function was used. The value of *S*_0_^2^ = 1 was fixed during the refinements.

Mössbauer absorption spectra were obtained on MS1104EM Express Mössbauer spectrometer (Cordon GmbH, Rostov-on-Don). The radiation source with an activity of 6 mCi was ^57^Co in a metal rhodium matrix (RITVERC GmbH, St. Petersburg, Russia). The spectra were obtained at room temperature (295 ± 3 K) and in a vacuumed cryostat at a liquid nitrogen temperature (77.5 ± 0.5 K). The spectra were collected until the signal to noise ratio was less than 1%. Mathematical processing was carried out for spectra with a high resolution (1,024 points) using SpectrRelax 2.4 (Lomonosov MSU, Russia) software. The isomer shift was determined relative to α Fe.

### Soil Pot Experiment

#### Fertilizers

A stock solution (1,000 μmol Fe L^-1^) of each ^57^Fe-humic NF (F, S, and M) at pH 7 was prepared from the freeze dried products (^57^Fe-NFs) previously obtained, as it was described above A stock solution of ^57^FeEDDHA (1,000 μmol Fe L^-1^) was prepared by chelation with Fe^3+^ from Fe(NO_3_)_3_ and *o-o*EDDHA [ethylenediamine-di (*o-o* hydroxyphenylacetic acid)] obtained from LGC Standards, Teddington, United Kingdom (93.12%), previously dissolved with three mol of NaOH per mol of chelating agent. The solution was adjusted at pH 7 with 1M KOH.

In order to test the ^57^Fe-NFs as correctors of iron chlorosis, three doses (35, 75, and 150 μmol ^57^Fe pot^-1^) were applied to iron deficient soybean plants and compared to ^57^FeEDDHA (50 μmol pot^-1^), as a positive control, and L (providing 8.9 μmol Fe pot^-1^). The treatments were applied over the soil surface 2 days after the soybean plants were transferred to the pots. Five replicates (five pots) per fertilizer were carried out.

#### Plant Material

Soybeans (*Glycine max AG1835* Asgrow Seed Co.) were germinated in the dark at room temperature on filter paper moistened with distilled water. After germination (7 days), seedlings were transferred to the growth chamber where they grew until the end of the experiment in a Dycometal-type CCK growth chamber provided with fluorescent and sodium vapor lamps with a 16 h, 25°C and 40% humidity day and 8 h, 20°C and 60% humidity night regime. Seedlings were placed on containers filled with 1/5 diluted nutrient solution of the full-strength solution with the following composition: macronutrients (mM) 1.0 Ca(NO_3_)_2_, 0.9 KNO_3_, 0.3 MgSO_4_, and 0.1 KH_2_PO_4_; cationic micronutrients (μM) 2.0 FeHBED, 2.5 MnSO_4_, 1.0 CuSO_4_, 10.0 ZnSO_4_, 1.0 CoSO_4_, 1.0 NiCl_2_, and 115.5 EDTANa_2_; anionic micronutrients (μM) 35.0 NaCl, 10.0 H_3_BO_3_, and 0.05 Na_2_MoO_4_ and 0.1 mM HEPES. The pH was adjusted to 7.5 with 1.0M KOH. After 8 days, the diluted nutrient solution was replaced by the full-strength solution without Fe. Seedlings were kept in this solution for 2 days in order to induce iron deficiency. In order to simulate calcareous conditions, CaCO_3_ (0.1 g L^-1^) was added to each pot. The deficient iron soybean plants (three plants per pot) were transferred into 600 g polystyrene pots filled in with the soil/sand 70/30% (w/w) mixture. The soil was obtained from the top 20 cm of a citrus farm at Picassent, Valencia, Spain (39°21′41.28^′′^ N, 0°27′42.58^′′^ W). Physicochemical characteristics of this soil are described in Table 1. Texture, pH, soil E.C., OM, C/N ratio, CaCO_3_ were measured according to the official methods (MAPA, 1994) and micronutrients availability was determined as described by Soltanpour and Schwab (1977). Normalized calcareous sand (2–4 mm) was used. One day before transferring the seedlings, pots were irrigated till field capacity. Water and iron free nutrient solution were added every day. Two samplings were carried out, at 15 and 48 days after the fertilizers (DAF) were applied.

**Table 1:** Physical and chemical properties of the soil used for both pot experiments.

Parameter	Picassent soil
^1^Sand (g ⋅ kg^-1^)	435
^1^Silt (g ⋅ kg^-1^)	80
^1^Clay (g ⋅ kg^-1^)	485
pH (H_2_O)	7.9
E.C._1:5_ (dS m^-1^)	2.0
^2^OM (g kg^-1^)	9.2
^3^N Kjeldahl (g kg^-1^)	0.3
C/N	30.7
^5^CaCO_3_ (g ⋅ kg^-1^)	380
^6^CaCO_3_ active (g ⋅ kg^-1^)	89
^8^DTPA Zn (mg ⋅ kg^-1^)	3.00
^8^DTPA Fe (mg ⋅ kg^-1^)	5.3
^8^DTPA Mn (mg ⋅ kg^-1^)	4.5
^8^DTPA Cu (mg ⋅ kg^-1^)	1.1


*E.C., electrical conductivity; OM, organic matter. ^*1*^Densitometry Bouyoucos’s method. ^*2*^Walkley-Black’s method. ^*3*^Kjeldahl’s method. ^*5*^Williams’s calcimeter. ^*6*^Droinean’s method. ^*7*^Exchangeable cations extracted with NH_*4*_Ac pH = 7. ^*8*^Soltanpour and Swab’s method.*

#### Analytical Procedures

The sampled roots, stems, and leaves were separated, weighted, and washed with 0.1% HCl and 0.01% non-ionic detergent (Tween 80) solutions, rinsed with distilled water (Álvarez-Fernández et al., 2001) and dried in a forced air oven at 65°C for 3 days. Thereafter, samples were milled and calcined in a muffle furnace (480°C). The ashes were digested using 7M HNO_3_ Suprapour.

Soil soluble fraction was obtained by washing the soil with distilled water (600 mL) by stirring for 10 min on a rotary shaker at 90 min^-1^. An aliquot of 40 mL was centrifuged at 9,000 min^-1^ for 10 min (Sorvall Legend XFR, Thermo Fisher Scientific, United States), the supernatant was first filtered with ashless filters paper (Grade 1238, Filter Lab) and then, through syringe cellulose filters (0.45 μm) (OlimPeak, Teknokroma). Nitric acid (Suprapur, Merck) was added to achieve a 1% acid matrix.

Soil available fraction was obtained from the solid residue in the centrifuge tube by extraction for 20 min with 25 mL using Soltanpour and Schwab (1977) extractant (DTPA + ammonium bicarbonate). After that, the samples were filtered. The extraction was made in triplicate, the extracts joined in a single extract, and volume made up to 100.0 mL. An aliquot of 7.165 mL of 65% HNO_3_ was added to eliminate the excess of bicarbonate and to allow an acid media for the analytical determinations.

Isotope quantification in the plant organs and soil fractions (soluble and available) were determined by ICP-MS (7500c, Agilent Technologies, Santa Clara, CA, United States) using ^57^Fe standards and correcting Ca and Ar interferences by means of a collision cell quadrupole ICP-MS instrument.

The specific contribution of each iron fertilizer to the soil and plant nutrition was calculated by isotope pattern deconvolution analysis considering the two iron sources, with a modification of the method proposed by Rodríguez-Castrillón et al. (2008) In brief, the mass balance for the Fe natural isotope can be expressed as shown by matrix notation:

[A54TotalA56TotalA57TotalA58Total]=[A54FerA54NatA56FerA56NatA57FerA57NatA58FerA58Nat]×[xFerxNat]+[e54e56e57e58]

where each *A_Total_* is the isotope abundance of each Fe isotope in the plant sample. *A_Fer_* is the corresponding isotope abundance in the tracer, and *A_Nat_* is the natural isotope abundance. Moreover, *x_Fer_* and *x_Nat_* denote the molar fractions of Fe in the isotopically altered sample arising from the two different sources of the element (fertilizer or natural). The best values of *x_Nat_* and *x_Fer_* are found by least-squares fitting of the error vector *e* (minimizing the square sum of errors) using the SOLVER tool in Excel^®^.

To evaluate the influence of Fe on leaf chlorophyll, the SPAD Index was measured every 2 or 3 days, using a Minolta Chlorophyll Meter SPAD-502 (Minolta, Osaka, Japan) after the first application of the Fe fertilizers.

### Statistical Analysis

In order to verify the homogeneity of the data, the Levene test was used first, prior to testing the differences between Fe treatments for significance by one-way ANOVA. Means were compared using the Duncan multiple range test (*P* < 0.05). Results of two-way ANOVA are expressed as ns (not significant), ^∗^*P* < 0.05, ^∗∗^*P* < 0.01, and ^∗∗∗^*P* < 0.001. All the calculations were performed using SPSS v.24.0 software.

## Results

### Characterization of ^57^Fe-NFs

The main characterization tools used in this work were^57^Fe Mössbauer spectroscopy, XRD, EXAFS, and TEM. They were applied to identify the iron phases in the ^57^Fe-NFs and to estimate the particle size. According to the XRD data, the major crystalline phases in all three samples of ^57^Fe-NFs are mineral salts of potassium and sodium: nitrates in the product F, and sulfates in the products S and M, the collected data are shown in Supplementary Figure SM2. No iron-containing crystalline phases were observed in the obtained XRD patterns. The XRD data on the synthesized reference samples – ferrihydrite and goethite – are shown in Supplementary Figure SM3.

**FIGURE 1:**
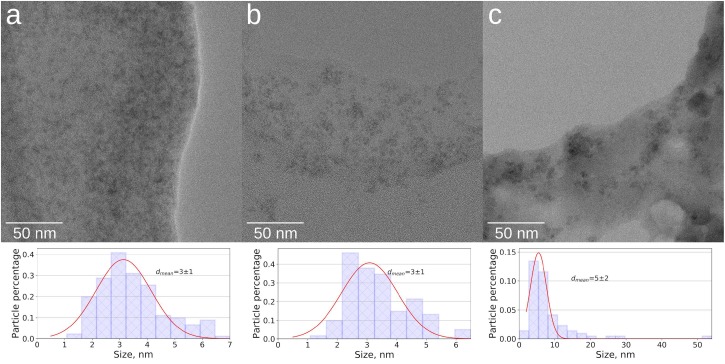
TEM images and size statistics for the three ^57^Fe-nanofertilizer samples under study: **(a)** F, **(b)** M, **(c)** S.

**FIGURE 2:**
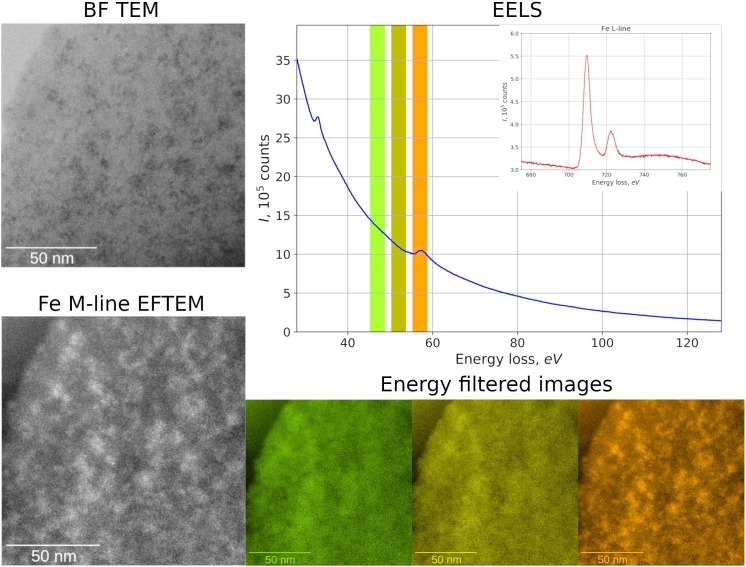
TEM, Fe M-line EFTEM, EELS spectra and the scheme of EFTEM measurements for the product F.

**FIGURE 3:**
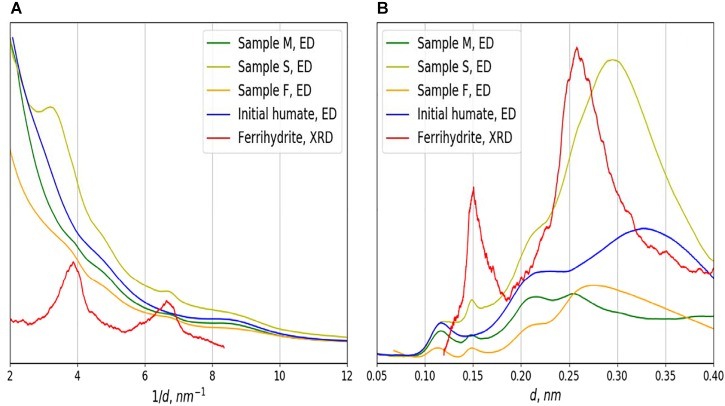
Results of ED integration in reciprocal space **(A)** and in direct space, after background removal **(B)** for the products F, M, S. and initial humate (L) in comparison with the XRD data for ferrihydrite.

TEM data demonstrate that all ^57^Fe-NFs samples contain large low density particles typical for HSs. Inside of these particles the nano-sized contrast variations can be observed, which correspond to the NPs with sizes < 5 nm (Figure 1a,b). Some aggregates of ∼20 nm were observed in the product S (Figure 1c). These NPs can be clearly seen in the zero loss images of the products F and M (Supplementary Figure SM3). Figure 2 shows TEM images, Fe M-line EFTEM, EELS spectra and the scheme of EFTEM measurements for the F sample, which was of particular interest for this study, because its synthesis was specifically run under conditions favoring formation of ferrihydrite phase. The Fe M- and L-lines were clearly observed in the EELS spectrum of this sample (Figure 2). This confirmed the presence of iron in the region of interest. The spatial distribution of iron was investigated using three-point EFTEM, which yielded the pattern of iron-containing NPs (bright inclusions) in the darker matrix (HSs). The obtained results are indicative of the iron-containing composition of the formed NPs. In order to investigate the structures of the observed NPs, the SAED patterns were measured (Supplementary Figure SM6) and compared to the ED of the parent HS (L) and XRD pattern of the synthesized pure two-line ferrihydrite (Ferrihydrite). ED patterns of all iron containing samples were characterized with appearance of two additional reflexes as compared to the parent L sample (Figure 3). The smaller peak was observed at 0.15 nm, and the larger one – at 0.25–0.30 nm. Such a combination of reflexes is similar to the two-line ferrihydrite diffraction pattern. However, for refining this assignment additional information was desirable and obtained with the use of XANES and EXAFS spectroscopy.

**FIGURE 4:**
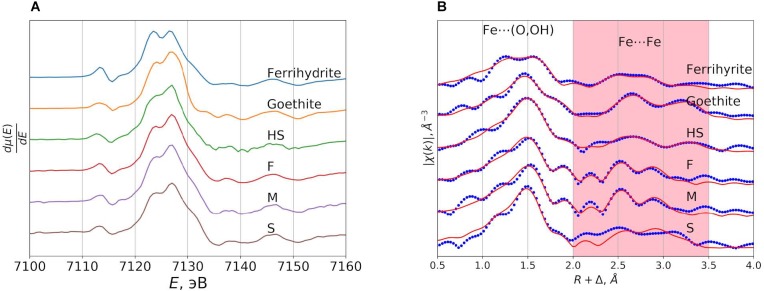
The first derivatives of the XANES spectra shown in Supplementary Figure SM7 **(A)** and the results of model approximation of the EXAFS spectra in R-space **(B)** for the ^57^Fe-labeled nanofertilizer samples under study.

The acquired XANES spectra of the samples obtained in this study are shown in Supplementary Figure SM7 and their first derivatives are presented in Figure 4A. The spectra obtained for all samples under study were rather similar and demonstrated the presence of ferric ions in octahedral coordination (Manceau and Gates, 1997). EXAFS spectra of the same samples (Figure 4B and Supplementary Figure SM8) were fitted to the two-shell model (Maillot et al., 2011). It assumes that the first shell contains two different oxygen positions (Fe-O), whereas the second one- three iron position (Fe–Fe) with the same value of σ^2^. The obtained results for ferrihydrite and goethite reference samples (Table 2) are in good agreement with the reported values for Fe–O and Fe–Fe distances (Maillot et al., 2011). For the samples under study, the values of Fe–Fe distances were very close to those of ferrihydrite (Table 2). For example, in the F sample (HS-ferrihydrite), they were 2.98 Å, 3.14 Å, and 3.53 Å, whereas in the reference sample – 2.91 Å, 3.06 Å, and 3.46 Å. These distances belong to face-sharing, edge-sharing, and corner-sharing octahedra in ferrihydrite, respectively (Manceau and Gates, 1997). Coordination number of iron at 2.91 Å and 3.06 Å for all three samples of the NFs are similar and indicative of ferrihydrite (Maillot et al., 2011). The high value of R-factor for the “S” sample results from the poorest quality of its approximation by the above two-shell model. This might be caused by heterogeneity of the S sample which contained different phases of iron (hydr)oxides. The iron in the parent humate resembles closely goethite phase. The differences in Fe–O distances between the reference ferrihydrite (1.82 Å and 1.97 Å) and the samples obtained in the presence of HS (1.94 Å and 2.11 Å) may be connected to very small size of the particles formed (<5 nm) and to respective surface effects. In the ideal ferrihydrite, the relation between tetrahedral Fe (IV Fe) and octahedral Fe (VI Fe) is 1:4. However, according to Michel et al. (2007), the size reduction of ferrihydrite particles leads to a decrease in IV Fe number. Moreover, Manceau and Gates (1997) demonstrated that 1.86Å Fe–O distance observed in the EXAFS spectra could not be directly assigned to the IV Fe due to the presence of short Fe–(O,OH) bonds on the surface. It means that the surface contamination and size reduction in the HS-stabilized ferrihydrite particles may lead to a decrease in a number of short Fe–O bonds as compared to the reference ferrihydrite. The applied EXAFS model involved only two Fe–O distances due to restriction on the amount of independent parameters, the most intense paths for each sample were chosen. The obtained data allow a conclusion that all three samples of the synthesized NFs were very similar with regard to the iron phase and contained iron (hydr)oxide with the polyhedral arrangement motif of the ferrihydrite.

**Table 2:** The model parameters calculated from the EXAFS spectra for three ^57^Fe-NFs samples used in this study.

Sample	*R*_f_ (%)	*ΔE* (eV)		*N*	R (Å)	σ^2^× 10^3^
“L”	1.5%	-0.9	O	2.7 ± 0.9	1.94 ± 0.02	2 ± 2
			O	1.3 ± 0.4	2.08 ± 0.04	
			Fe	2.1 ± 0.8	3.05 ± 0.03	10^∗^
			Fe	1.8 ± 1.2	3.24 ± 0.06	
			Fe	2.4 ± 0.9	3.48 ± 0.03	
Ferrihydrite	2.9%	-7	O	1.2 ± 0.4	1.82 ± 0.04	3 ± 3
			O	2.3 ± 0.8	1.97 ± 0.02	
			Fe	0.5 ± 0.3	2.91 ± 0.04	0 ± 6
			Fe	0.5 ± 0.4	3.06 ± 0.04	
			Fe	0.3 ± 0.2	3.46 ± 0.04	
Goethite	1.5%	-0.1	O	2.7 ± 0.6	1.94 ± 0.02	3 ± 2
			O	2.2 ± 0.4	2.09 ± 0.02	
			Fe	3.3 ± 1.4	3.07 ± 0.02	9 ± 3
			Fe	2.8 ± 1.6	3.30 ± 0.04	
			Fe	3.6 ± 1.3	3.47 ± 0.03	
“F”	3.0%	0.3	O	2.6 ± 0.8	1.94 ± 0.02	3 ± 3
			O	1.3 ± 0.4	2.11 ± 0.04	
			Fe	0.7 ± 0.4	2.98 ± 0.02	0 ± 4
			Fe	0.9 ± 0.5	3.14 ± 0.03	
			Fe	0.1 ± 10.2	3.49 ± 0.3	
“M”	2.9%	0.2	O	2.8 ± 1.1	1.94 ± 0.02	3 ± 3
			O	1.1 ± 0.5	2.10 ± 0.0C	
			Fe	0.9 ± 0.5	2.97 ± 0.02	0 ± 3
			Fe	0.9 ± 0.5	3.13 ± 0.03	
			Fe	0.2 ± 0.2	3.53 ± 0.09	
“S”	4.7%	3.2	O	1.4 ± 0.3	1.95 ± 0.02	0.5 ± 2.5
			O	2.0 ± 0.5	2.10 ± 0.03	
			Fe	0.9 ± 0.7	3.01 ± 0.03	2 ± 7
			Fe	1.1 ± 0.8	3.16 ± 0.04	
			Fe	0.4 ± 0.5	3.51 ± 0.07	


*^∗^ Fixed value.*

**FIGURE 5:**
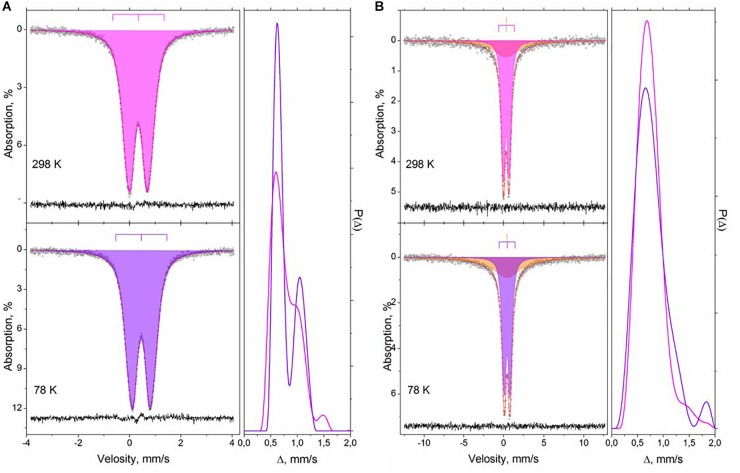
Mössbauer spectra of the M and S samples (**A,B**, respectively) recorded at 295K and 78K and the quadrupole splitting distributions for these spectra.

The results of Mössbauer spectroscopy investigation yielded additional support to this conclusion. The Mössbauer spectra of M and F samples recorded at room temperature and at liquid nitrogen showed broadened electric quadrupole doublets as it is demonstrated in Figure 5A on the example of M-sample. The Mössbauer spectra of F-sample is shown in Supplementary Figure SM11. This could be explained by the distribution of gradients of the electric field (Supplementary Figure SM10). For these samples, the distribution of quadrupole splittings has a bimodal character, similar to the distribution for high-temperature spectra of a reference sample of ferrihydrite (Supplementary Figure SM10). The small size of the particles under study and the low blocking temperature might be the reasons for a lack of substantial broadening of the resonance lines as well as the absence of a magnetically ordered fraction in the spectra at low temperature. Even for the reference sample of ferrihydrite with the larger particles as compared to the NPs stabilized by humate, the magnetic ordering (Supplementary Figure SM10) was not observed at the boiling point of liquid nitrogen (Schwertmann et al., 1999).

The spectra of S sample at these temperatures, in addition to very similar quadrupole doublets, contained extended absorption with low intensity, which can be conditionally described by a singlet line of a large width (Figure 5B). The relative area of extended absorption in the S sample did not change with temperature indicating that it is not related to superparamagnetism. This can be connected with weak spin–spin interactions between iron (III) atoms in strongly inhomogeneous and disordered medium. These interactions could be precisely observed because of the use of ^57^Fe (Pankratov et al., 2013). Distribution of quadrupole splitting in the S sample is unimodal and it has significantly larger dispersion as compared to those in the samples M and F. This might be indicative of the larger diversity of the local environments of iron atoms in the S sample. The further details on Mössbauer data can be found in the Supplementary Table SM1 and Supplementary text. In general, the data of Mössbauer spectroscopy are in good agreement with the data of TEM and X-ray spectroscopy.

### Soil Pot Experiment

In order to evaluate the ^57^Fe-NFs effect in the plant growth, the SPAD index and the fresh weight of soybean shoots and roots at 48 DAF were measured and presented in Table 3. There was no leaf chlorosis observed and the plants presented SPAD indexes > 25, which is indicative of sufficient iron nutrition (Martín-Fernández et al., 2017b). The shoot biomass of the fertilized plants (in case of F3, S1, S2, S3, and M2) was larger as compared to FeEDDHA. There was no significant differences observed in the root biomass of the plants fertilized with the NFs under study (except for F3, which showed the largest fresh weight of roots). In our former studies, the plants fertilized with the leonardite humates accumulated slightly higher fresh weight than those fertilized with the iron chelate. According to Rose et al. (2014), the HSs generally increase the shoot and root growth by 15–25%. Canellas et al. (2015) reported that the growth response of monocotyledonous plants to exogenously applied HS is more sensitive as compared to for dicotyledonous plants, and the plant physiological responses to HS isolated from brown coal (e.g., lignite, leonardite, and subbituminous coals) are less than those observed in response to the addition of HS isolated from peat, composts or vermicomposts.

**Table 3:** SPAD index at the last level of trifoliate well developed soybean leaves and fresh weight (FW) of shoots and roots of soybean control (L) plants and plants fertilized with the ^57^Fe products F, S, and M in three doses: (1) 35, (2) 75, and (3) 150 μmol ^57^Fe pot^-1^ or 50 μmol ^57^FeEDDHA pot^-1^ at 48 DAF.

Treatments	SPAD	Shoot FW (g pot^-1^)	Root FW (g pot^-1^)
L	41.8 ± 2.04^abc^	13.9 ± 0.47^a^	5.69 ± 0.62^b^
F1	37.1 ± 1.55^cd^	13.4 ± 0.50^ab^	7.33 ± 0.61^ab^
F2	39.2 ± 1.03^bc^	13.8 ± 0.09^a^	6.90 ± 0.22^ab^
F3	45.9 ± 0.50^ab^	14.6 ± 0.39^a^	8.15 ± 0.60^a^
S1	40.2 ± 1.75^bc^	14.0 ± 0.73^a^	6.80 ± 0.40^ab^
S2	38.4 ± 1.98^bc^	14.5 ± 0.55^a^	7.03 ± 0.97^ab^
S3	30.8 ± 4.75^d^	13.9 ± 0.48^a^	6.61 ± 0.32^ab^
M1	39.1 ± 3.70^bc^	13.4 ± 0.16^ab^	6.81 ± 0.54^ab^
M2	42.4 ± 2.84^abc^	13.7 ± 0.45^a^	6.34 ± 0.25^ab^
M3	40.6 ± 2.44^bc^	13.3 ± 1.13^ab^	7.22 ± 0.79^ab^
FeEDDHA	48.9 ± 1.53^a^	11.5 ± 0.74^b^	5.70 ± 0.45^b^


*For each series different letters denote significant differences among the treatments according to Duncan’s Test (*p* < 0.05). Results are expressed as averages ± standard error, *n* = 5.*

The ^57^Fe tracer technique allowed monitoring Fe from the fertilizer (Fe_Fer_) in the soil experiment and distinguishing it from the native Fe contained in the soil (Fe_Nat_). The Fe_Total_ was calculated as the sum of Fe_Fer_ and Fe_Nat_. A combination of ^57^Fe isotope and mathematical deconvolution was a relevant tool to evaluate the efficacy between different NFs to correct iron deficiency.

**FIGURE 6:**
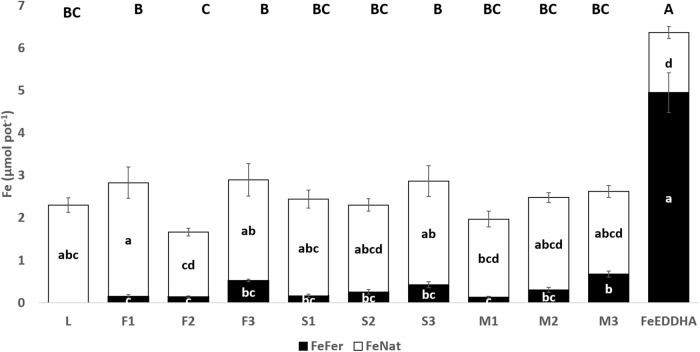
Fe_Fer_ and Fe_Nat_ (μmol pot^-1^) contents in soybean shoots of control plants (L) and plants fertilized with the ^57^Fe products F, S, and M in three doses: (1) 35, (2) 75, and (3) 150 μmol ^57^Fe pot^-1^ or 50 μmol ^57^FeEDDHA pot^-1^, calculated as the sum of the first (15 DAF) and second sampling (48 DAF). For each series different letters denote significant differences among the treatments according to Duncan’s Test (*p* < 0.05). Lowercase letters correspond to Fe_Fer_ and Fe_Nat_ and capital letters correspond to Fe_Total_ statistical results. Results are expressed as averages ± standard error, *n* = 5.

The contents of Fe_Fer_, Fe_Nat_, and Fe_Total_ (μmol pot^-1^) in soybean shoots were calculated as the sum of the first (15 DAF) and second samplings (48 DAF) and presented in Figure 6. The effect of type and doses of the ^57^Fe-NFs on the contents of Fe_Fer_, Fe_Nat_ and Fe_Total_ in soybean shoots was studied by ANOVA two-way statistical analysis and presented in Table 4. Significant differences were observed between ^57^Fe-NFs types and doses. The product M provided the highest Fe_Fer_ content to the soybean shoots mainly when the third dose was applied (150 μmol ^57^Fe pot^-1^). The content of Fe_Total_ expressed as the sum of the Fe_Fer_ and Fe_Nat_ showed significant differences only between the doses, and the third dose seemed to be the most adequate. Table 5 presents the Fe_Total_ (mg kg^-1^) concentration in soybean leaves at 15 and 48 DAF. It ranged from 36.8 mg kg^-1^ (F1) to 53.6 mg kg^-1^ (M1) at 15 DAF and from 34.6 mg kg^-1^ (S1) to 39.9 (S3) mg kg^-1^ at 48 DAF. According to the iron concentration obtained in a previous work (Rodríguez-Lucena et al., 2010) and the SPAD index detected (Table 4), the soybean plants are sufficiently iron nourished and did not present symptoms of iron chlorosis. Moreover, the iron concentration in leaves decreased for the second sampling because iron was a priority for the pods production, even for plants fertilized with ^57^FeEDDHA.

**Table 4:** Effect of doses (D) and nanofertilizers (NFs) related to the contents of Fe_Fer_, Fe_Nat_, and Fe_Total_ (μmol pot^-1^) in soybean shoots, roots, pods, soluble and available soil fraction for soybean plants fertilized with the products F, S, and M with three doses (35, 75, and 150 μmol pot^-1^) at 48 DAF.

					Nanofertilizers			Doses (μmol pot^-1^)		
						
		D	NFs	DxNFs	F	S	M	35	75	150
**Shoots**	**Fe_Fer_**	^∗∗∗^	^∗^	^∗^	0.27 ± 0.03^b^	0.28 ± 0.03^b^	0.35 ± 0.03^a^	0.15 ± 0.03^c^	0.23 ± 0.03^b^	0.54 ± 0.03^a^
	**Fe_Nat_**	ns	ns	ns	2.19 ± 0.15	2.26 ± 0.15	1.99 ± 0.15^ns^	2.27 ± 0.15	1.92 ± 0.15	2.25 ± 0.15^ns^
	**Fe_Total_**	^∗^	ns	ns	2.44 ± 0.16	2.53 ± 0.16	2.36 ± 0.16^ns^	2.41 ± 0.16^ab^	2.15 ± 0.16^b^	2.77 ± 0.16^a^
**Pods**	**Fe_Fer_**	^∗∗∗^	^∗^	ns	0.20 ± 0.04^b^	0.19 ± 0.16^b^	0.34 ± 0.16^a^	0.11 ± 0.04^c^	0.22 ± 0.04^b^	0.40 ± 0.04^a^
	**Fe_Nat_**	ns	ns	ns	0.94 ± 0.09	0.97 ± 0.09	1.08 ± 0.09^ns^	1.15 ± 0.09	0.94 ± 0.09	0.90 ± 0.09^ns^
	**Fe_Total_**	ns	^∗^	^∗^	1.14 ± 0.10^b^	1.14 ± 0.11^b^	1.42 ± 0.10^a^	1.27 ± 0.10	1.22 ± 0.10	1.27 ± 0.10^ns^
**Roots**	**Fe_Fer_**	^∗∗∗^	ns	ns	1.64 ± 0.29	1.21 ± 0.29	1.15 ± 0.29^ns^	0.45 ± 0.30^b^	0.75 ± 0.29^b^	2.80 ± 0.27^a^
	**Fe_Nat_**	ns	ns	ns	77.7 ± 8.23	71.8 ± 8.23	77.8 ± 8.23^ns^	67.6 ± 8.23	74.4 ± 8.23	85.2 ± 8.23^ns^
	**Fe_Total_**	ns	ns	ns	79.4 ± 8.34	73.1 ± 8.34	79.0 ± 8.34^ns^	68.2 ± 8.34	75.2 ± 8.34	88.0 ± 8.34^ns^
**Soluble**	**Fe_Fer_**	^∗∗^	^∗^	^∗^	0.16 ± 0.11^b^	0.20 ± 0.11^b^	0.53 ± 0.10^a^	0.18 ± 0.10^b^	0.06 ± 0.11^b^	0.65 ± 0.11^a^
	**Fe_Nat_**	ns	ns	ns	5.24 ± 3.49	9.80 ± 3.49	15.6 ± 3.49^ns^	10.6 ± 3.49	6.19 ± 3.49	13.8 ± 3.49^ns^
	**Fe_Total_**	ns	ns	ns	5.39 ± 3.43	10.0 ± 3.57	16.2 ± 3.43^ns^	10.8 ± 3.43	6.36 ± 3.43	14.5 ± 3.57^ns^
**Available**	**Fe_Fer_**	^∗∗∗^	^∗∗∗^	^∗∗∗^	27.3 ± 2.51^a^	10.3 ± 2.51^c^	17.7 ± 2.51^b^	7.83 ± 2.51^a^	17.3 ± 2.51^b^	30.1 ± 2.51^c^
	**Fe_Nat_**	ns	^∗^	ns	179 ± 55.8^b^	169 ± 58.1^b^	364 ± 55.8^a^	255 ± 55.8	212 ± 55.8	245 ± 58.1^ns^
	**Fe_Total_**	ns	^∗^	ns	207 ± 56.8^b^	179 ± 59.2^b^	382 ± 56.8^a^	263 ± 56.8	230 ± 56.8	274 ± 59.2^ns^


*Means (*n* = 5) in the same row followed by the same letter do not differ significantly according to the Duncan test (*P* < 0.05). Two-way ANOVA results. ^∗^*P* < 0.05, ^∗∗^*P* < 0.01, ^∗∗∗^*P* < 0.001; ns, not significant.*

**Table 5:** Fe_Total_ (mg kg^-1^) concentration in soybean leaves and pods of plants fertilized with the ^57^Fe products F, S, and M in three doses: (1) 35, (2) 75, and (3) 150 μmol ^57^Fe pot^-1^ or 50 μmol^57^FeEDDHA pot^-1^ at 15 and 48 DAF and soybean pods at 48 DAF.

Treatments	Leaves 15 DAF	Leaves 48 DAF	Pods
L	38.1 ± 2.85^d^	48.3 ± 5.65^b^	49.5 ± 5.40^ab^
F1	36.8 ± 1.10^d^	38.6 ± 3.52^bc^	46.1 ± 9.53^ab^
F2	41.3 ± 3.38^cd^	37.5 ± 2.60^bc^	32.6 ± 6.85^b^
F3	40.2 ± 3.61^d^	39.4 ± 2.40^bc^	38.9 ± 4.00^b^
S1	38.2 ± 3.11^d^	34.6 ± 1.43^c^	45.7 ± 5.38^ab^
S2	38.9 ± 2.73^d^	35.8 ± 1.97^c^	45.7 ± 5.58^ab^
S3	38.1 ± 2.59^d^	39.9 ± 1.81^bc^	45.9 ± 3.26^ab^
M1	53.6 ± 4.29^b^	35.4 ± 0.53^c^	51.3 ± 2.90^ab^
M2	46.1 ± 1.20^bcd^	35.5 ± 2.61^c^	57.9 ± 7.43^a^
M3	50.7 ± 3.11^bc^	39.5 ± 2.16^bc^	49.8 ± 3.63^ab^
FeEDDHA	139 ± 5.60^a^	108 ± 6.99^a^	46.7 ± 3.13^ab^


*For each series different letters denote significant differences among the treatments according to Duncan’s Test (*p* < 0.05). Results are expressed as averages ± standard error, *n* = 5.*

**FIGURE 7:**
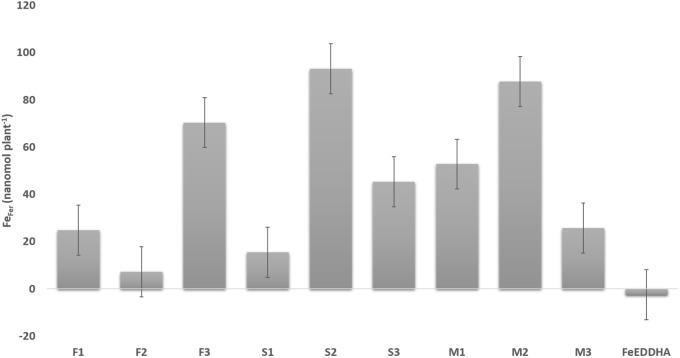
Differences in Fe_Fer_ uptake (%) in soybean leaves of plants fertilized with the ^57^Fe products F, S, and M in three doses: (1) 35, (2) 75, and (3) 150 μmol ^57^Fe pot^-1^ or 50 μmol ^57^FeEDDHA pot^-1^, between the first (15 DAF) and the second sampling (48 DAF). Results are expressed as averages ± standard error, *n* = 5.

Differences in Fe_Fer_ uptake (nmol plant^-1^) in soybean leaves between the first (15 DAF) and the second samplings (48 DAF) were calculated and plotted in Figure 7. The plants fertilized with the ^57^Fe-NFs, nominally, with S2, M2 and F3, have taken up 93, 88, and 70 nmol Fe_Fer_ plant^-1^ in leaves in 33 days, whereas the plants fertilized with FeEDDHA stopped providing Fe_Fer_ to the leaves after the first sampling. The products S and M, prepared from ^57^Fe_2_(SO_4_)_3_, at their second dose (75 μmol ^57^Fe pot^-1^) and the product F, prepared from ^57^Fe(NO_3_)_3_ at the third dose (150 μmol ^57^Fe pot^-1^) showed the highest Fe_Fer_ increase between sampling times. These results are consistent with our previous data (Cieschi et al., 2017) on fertilization with iron leonardite humate which sustained slow and increasing iron nutrition to citrus growth under conditions of calcareous soil and yielded results similar to FeEDDHA with regard to efficacy of iron deficiency correction during the first year of application.

**FIGURE 8:**
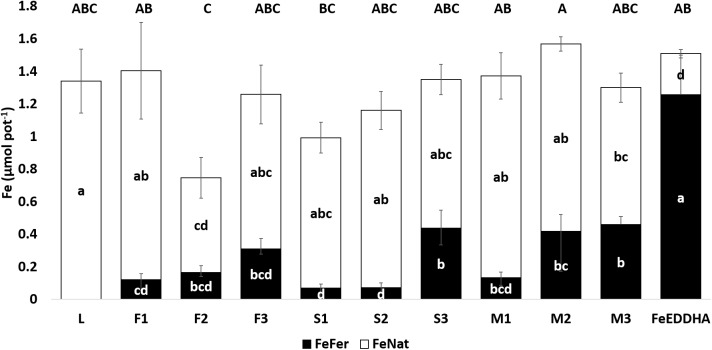
Fe_Fer_ and Fe_Nat_ (μmol pot^-1^) contents in soybean pods of control plants (L) and plants fertilized with the ^57^Fe products F, S, and M in three doses: (1) 35, (2) 75, and (3) 150 μmol ^57^Fe pot^-1^ or 50 μmol ^57^FeEDDHA pot^-1^ at 48 DAF. For each series different letters denote significant differences among the treatments according to Duncan’s Test (*p* < 0.05). Lowercase letters correspond to Fe_Fer_ and Fe_Nat_ and capital letters correspond to Fe_Total_ statistical results. Results are expressed as averages ± standard error, *n* = 5.

Of particular interest are the contents of Fe_Fer_, Fe_Nat_ and Fe_Total_ (μmol pot^-1^) in soybean pods at 48 DAF, which are presented in Figure 8. The Fe_Fer_ content in pods increased along with an increase in the dose of ^57^Fe-NFs. The significant differences were observed between the doses and the ^57^Fe-NF type when the obtained results were compared using ANOVA two-way statistical analysis (Table 4). As in case of the shoots, the product M provided the higher content of Fe_Fer_ as compared to the other two ^57^Fe-NFs to the soybean pods and the third dose was the most efficient. Moreover, the similar Fe_otal_ contents in soybean pods were observed for the plants treated with ^57^Fe-NFs (except for F2) or FeEDDHA, and the product M was the most efficient in providing Fe_Total_ to the soybean pods regardless of the dose applied. Table 5 shows the Fe_Total_ concentration (mg kg^-1^) in soybean pods at 48 DAF. It ranged from 32.6 mg kg^-1^ (F2) to 57.8 mg kg^-1^ (M2) for the plants fertilized with ^57^Fe-NFs. According to Römheld and Nikolic (2007), the accumulation of total iron in pods for soybean plants reaches 50 mg kg^-1^ under conditions of sufficient nourishment. Nadal et al. (2012) and Martín-Fernández et al. (2017a) have observed ^57^Fe in soybean fruit of plants fertilized with *o,o*EDDHA/^57^Fe^3+^ and HBED/^57^Fe^3+^. Still, this study is the first one when ^57^Fe supplied by iron humates was detected in soybean pods. Shoots of plants fertilized with FeEDDHA showed the highest Fe_Fer_, the lowest Fe_Nat_ and the highest Fe_Total_ contents. The pods showed similar results for Fe_Fer_ and Fe_Nat_ contents.

**FIGURE 9:**
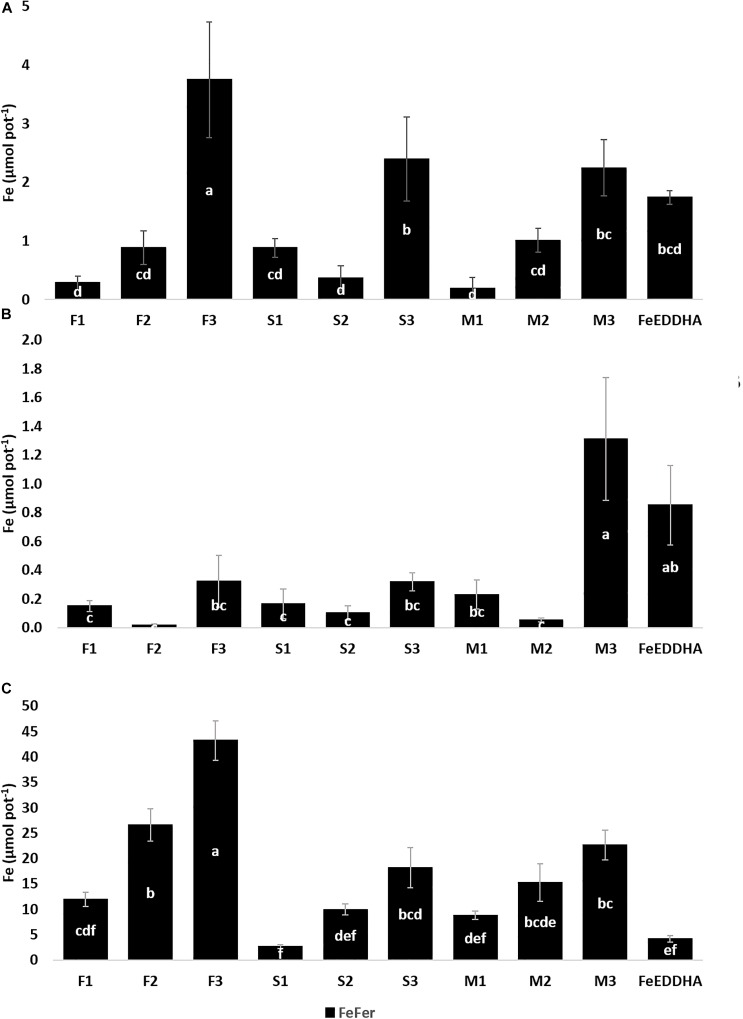
Fe_Fer_ (μmol pot^-1^) content in soybean roots of plants fertilized with the ^57^Fe products F, S, and M in three doses: (1) 35, (2) 75, and (3) 150 μmol ^57^Fe pot^-1^ or 50 μmol ^57^FeEDDHA pot^-1^ **(A)**, soluble soil fraction **(B)**, and available soil fraction **(C)** at 48 DAF. For each series different letters denote significant differences among the treatments according to Duncan’s Test (*p* < 0.05). Results are expressed as averages ± standard error, *n* = 5.

The content of Fe_Fer_ (μmol pot^-1^) in soybean roots as well as the contents of soluble and available soil fractions at 48 DAF are presented in Figure 9. In general, an increase in the content of Fe_Fer_ in soybean roots was observed when the plants were treated with the ^57^Fe-NFs (Figure 9A). The significant differences between the doses were confirmed by the ANOVA two-way statistical analysis (Table 4). The third dose was the most prone to store Fe_Fer_ in roots, in particular, in case of the F sample.

The Fe_Fer_ content in the soluble soil fraction was increasing along with the dose of the ^57^Fe-NFs. The results obtained for F3, S3, M3, and FeEDDHA were similar, though the highest Fe_Fer_ content was observed in the pots treated with M3 (Figure 9B). The ANOVA two-way statistical analysis has confirmed these results when the effect of the ^57^Fe-NF type and doses were compared (Table 4). Relating to the Fe_Fer_ content in the available soil fraction, the pots fertilized with the product F differed substantially from the others, in particular, in case of the third dose (Figure 9C and Table 4). In general, the Fe_Fer_ from the product F at the highest dose (150 μmol ^57^Fe pot^-1^) remained mostly available in soil (Figure 9C) or in the roots (Figure 9A).

**FIGURE 10:**
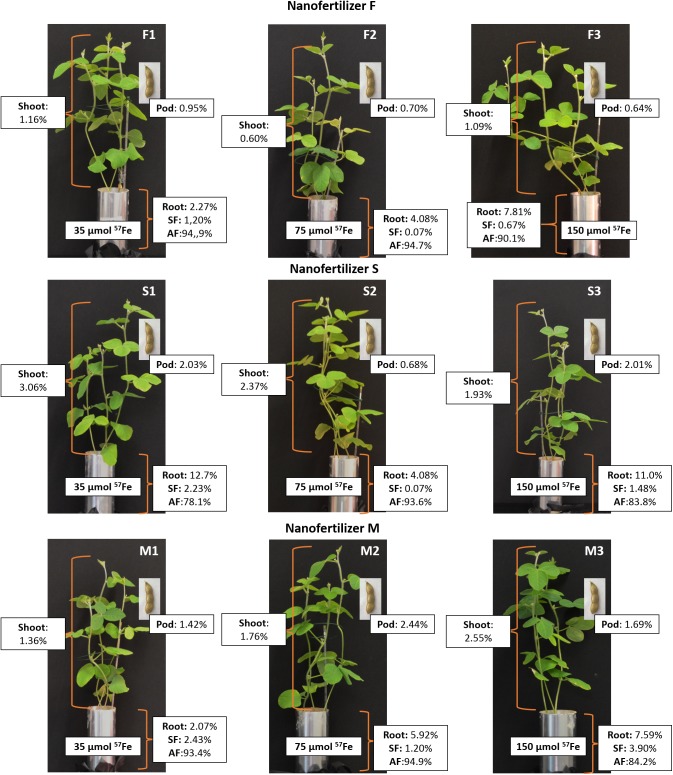
Distribution of ^57^Fe (%) in soybean shoots, pods and roots of plants fertilized with the ^57^Fe products F, S, and M in three doses: (1) 35, (2) 75, and (3) 150 μmol ^57^Fe pot^-1^ and in the soluble and available fraction soil.

Figure 10 shows the ^57^Fe (%) distribution in soybean plants (shoots, pods and roots) of plants fertilized with the ^57^Fe products F, S and M in three doses (35, 75 and 150 μmol ^57^Fe pot^-1^) and in the soil (soluble and available fraction). In general, the ^57^Fe-NFs remained in soil and ranged from 80% (S1) to 95% (F1), mainly in the available soil fraction. With respect to the plant, the highest percentage of ^57^Fe was detected in plants fertilized with S1 (18%), but mainly in roots. The ^57^Fe content in shoots increased along with the dose for plants fertilized with F and M.

## Discussion

In our work, three ^57^Fe-NFs (F, M, and S) were obtained and exhaustively characterized by XRD, TEM with ED, EELS and EFTEM, X-ray absorption spectroscopy (XANES and EXAFS) and Mössbauer spectroscopy. There were similarities among them with regard to the iron phase and iron (hydr)oxide content with the polyhedral arrangement motif of the ferrihydrite. Most agricultural soils contain natural ferrihydrite NPs, which may contribute to iron nutrition of plants. Many authors (Römheld and Nikolic, 2007; Colombo et al., 2012; Cieschi et al., 2017) reported ferrihydrite formation during the iron humate synthesis, they characterized and studied the relationship between the particle size, pH and stability. Angelico et al. (2014) and Colombo et al. (2015) have shown that the phase of iron (hydr)oxide formed in the presence of HS depends on pH, oxidation rate, and Fe:HS ratio.

Our pot experiments revealed that the ^57^Fe-NFs were capable of supplying ^57^Fe to the plants and it was transported from root to shoot and reached the pods (Figure 6, 8, 9A). In particular, we have observed that the plants fertilized with the product M presented the highest contents of ^57^Fe in shoots, pods and the soil soluble fraction, according to the two-way ANOVA statistical analysis (Table 4). This iron humate was prepared taking into account its maximum complexing capacity in order to avoid the iron flocculation in calcareous conditions. Then, the Fe:HS ratio obtained after the synthesis was the lowest (0.12 g Fe g org. C^-1^) which suggested that the high content of HS has stabilized the poorly ordered ^57^Fe structures entrapped into humic matrix and favored the iron uptake by the soybean plants. Similar results were obtained by Kulikova et al. (2017) for their product Fe-HA (4% Fe, 68% HA) which was tested with wheat plants grown under hydroponic conditions.

In soil, the ^57^Fe-NFs presented an increasing tendency to remain available to the plant requirements for the different growth stages (Figure 9C). In addition, the slow and continuous iron release from these NFs has confirmed their long-term effect in providing iron in calcareous conditions in contrast to the short-term effect of the iron synthetic chelate (Figure 7), reported in the previous studies (Cieschi et al., 2017). Moreover, in a recent hydroponic assay (Cieschi and Lucena, 2018), plants fertilized with FeEDDHA presented the highest Fe contents in roots after 10 days but at longer term exposition (60 days) of the plants treated with iron humates yielded iron uptake similar to the plants fertilized with the iron synthetic chelate. We hypothesized that an increase in iron humate concentration in the rhizosphere might cause a decrease in the transcription level of the genes involved in the iron transport and shoot growth, and so the iron transport from root to shoot decelerated. Several authors (Aguirre et al., 2009; Tomasi et al., 2013; Olaetxea et al., 2015; Zamboni et al., 2016) suggested that the efficiency of the root transcriptional response to Fe supply depends on the nature (physicochemical characteristics) of the ligand and its capability to activate Fe uptake mechanisms and translocations. In particular, Zamboni et al. (2016) demonstrated that Fe complexed to water-extractable HSs from peat did not cause relevant changes in the root transcriptome of tomato plants with respect to Fe-deficient plants. However, Aguirre et al. (2009) observed that high doses of a purified humic acid from leonardite applied to cucumber plants promoted the upregulation of CsFRO1 and CsIRT1 gene expression for 48 and 72 h while these genes were downregulated for 96 h. The authors suggested that it may be associated to root iron accumulation and/or iron translocation. Olaetxea et al. (2018) proposed that it is very likely that the action of HS on plant mineral nutrition involves a coordinated functional crosstalk between indirect and direct HS effects on the soil–plant system. Soil and, in particular, the rhizosphere are extremely complex environments with a large degree of heterogeneity down to the nanoscale where the interactions between soil constituents, plant roots, and microorganisms take place (Mimmo et al., 2014). Thus, the long-term effect would be an expected result.

With respect to the uptake of nanoparticles by plants, Kulikova et al. (2014) have observed that particles of HA were transferred from root to shoot of wheat seedlings through the plant vascular system and Nardi et al. (2002) have previously observed the same mechanism for low molecular weight fraction of HA (<2.5 kDa). Furthermore, Pariona et al. (2016) have recently detected by 3D microscopic techniques, clusters of hematite and ferrihydrite NPs in endodermis, xylem, phloem vessels, and cell walls of the xylem vessels of maize stems of plants grown in hydroponic conditions. The extensive studies of R. Pinton and S. Cesco’s group (Cesco et al., 2000, 2002; Nikolic et al., 2003; Colombo et al., 2012, 2014; Tomasi et al., 2013; Mimmo et al., 2014) modeled variety of interactions in soils of iron humate complexes according to the molecular weight of HS. They have demonstrated that low-molecular weight HS can form soluble complexes of Fe and move toward the root, acting as natural substrates for the membrane Fe (III)-chelate reductase and stimulate the proton release promoting the Fe acquisition for Strategy I plants. Recently, Homonnay et al. (2016) have carried out a preliminary study about iron nanoparticles in plant nutrition and hypothesized that it would be possible to consider for plant nutrition supply in the soil iron-based oxide or oxy-hydroxide nanoparticles since storage of iron in cells usually implies formation of ferritin which has a certain similarity with ferrihydrite/ferric hydrous oxide nanoparticles with variable amounts of phosphate.

Further research is needed to redesign the classical model of the iron uptake by plant with more studies that consider uptake from the Fe-NPs.

## Conclusion

According to Lal (2008), in the context of sustainable agriculture, applying innovative nanotechnology in agriculture is regarded as one of the promising approaches to significantly increase crop production. The Fe-NFs can be considered as a part of a novel technology in line with the precision and sustainable agriculture. They are iron-natural complex NPs synthetized from leonardite and they contain ferrihydrite in their structures which was properly and widely characterized. Moreover, the ^57^Fe-NFs used in this paper are capable of supplying Fe to the plants, transport it from root to shoot and reach the soybean pods. The slow and continuous iron release of these ^57^Fe-NFs confirms their long-term effect in providing iron in calcareous conditions while in soil, they tends to remain available to the plant requirements for the different growth stages.

Although further research is needed about the contribution of iron nanoparticles in plant nutrition, the Fe-NFs offers a natural, low cost and environmental option to the traditional iron fertilization in calcareous soils.

## Author Contributions

MC: synthesis of ^57^Fe-NFs, carried out of the soil experiment, data processing, and manuscript preparation. AP: synthesis of ^57^Fe-NFs and manuscript preparation. VL: XRD, TEM, and EXAFS data processing. DV: ICP AES analysis of ^57^Fe-NFs samples. DP: Mössbauer spectroscopy. AV: EXAFS spectroscopy. IP: design and supervision of the synthesis and characterization of the ^57^Fe-NFs, data interpretation, and manuscript preparation. JL: design and supervision of the soil experiment, data interpretation, and manuscript preparation.

## Conflict of Interest Statement

The authors declare that the research was conducted in the absence of any commercial or financial relationships that could be construed as a potential conflict of interest.

## Abbreviations

^57^Fe-NFs: iron-humic nanofertilizers isotopically labeled
ANOVA: analysis of variance
DAF: days after fertilizers applications
DTPA: diethylenetriaminepentaacetic acid
E.C.: electrical conductivity
ED: electron diffraction
EELS: electron energy loss spectra
EXAFS: extended X-ray absorption fine structure spectroscopy
Fe-MCC: iron-maximum complexing capacity
Fe-Nps: iron nanoparticles
FeHBED: iron (III) *N*,*N*’-bis(*o*-hydroxybenzyl)-ethylenediamine-*N*,*N*’-diacetic acid complex
HEPES: *N*-(2-hydroxyethyl) piperazine *N*’-(2-ethanesulfonic acid)
HS: humic substances
ICP-OES: inductively coupled plasma – optical emission spectrometry
ICP-MS: inductively coupled plasma mass spectrometry
L: leonardite potassium humate
NFs: nanofertilizers
*o-o*EDDHA: ethylenediamine-di (*o-o* hydroxyphenylacetic acid)
OM: soil organic matter
SAED: selected area electron diffraction
SPAD: soil–plant analysis development
TEM: transmission electron microscopy
XANES: X-ray absorption near edge structure
XAS: X-ray absorption spectra
XRD: X-ray diffraction
