# Supplementary material for: Eco-Friendly Iron-Humic Nanofertilizers Synthesis for the Prevention of Iron Chlorosis in Soybean (Glycine max) Grown in Calcareous Soil
Source: Front Plant Sci. 2019 Apr 5;10:413. doi: 10.3389/fpls.2019.00413 (PMC6460895; doi:10.3389/fpls.2019.00413)
Supplement: Supplementary file 1 [file Data_Sheet_1.doc]

**Supplementary Materials**

**Iron-humic nanofertilizers obtained by directed synthesis to provide iron in calcareous soil**

María T. Cieschi^1^, Alexander Yu. Polyakov^2, 3^, Vasily A. Lebedev^4^, Dimitry S. Volkov^4, 6^, Denis A. Pankratov^4^, Alexey A. Veligzhanin^5^, Irina V. Perminova^4*^, Juan J. Lucena^1*^

^1^Autonomous University of Madrid. Department of Agricultural Chemistry and Food Science. Madrid. Spain.

^2^Kurnakov Intitute of General and Inorganic Chemistry. Russian Academy of Sciences, Moscow. Russia

^3^Department of Materials Science, Lomonosov Moscow State University, Moscow. Russia

^4^ Department of Chemistry, Lomonosov Moscow State University Moscow. Russia

^5^National Research Center “Kurchatov Institute”, Akademik Kurchatov Square 1, 123098 Moscow, Russia

^6^ V.V. Dokuchaev Soil Science Institute, Moscow, Russia

**Determination of the Fe-MCC**

Maximum complexing capacity corresponds to the maximum iron content, Fe (III) that can be bound to the potassium humate structure without clotting. To determine the Fe-MCC of the potassium humate, based on the work of Villén et al*.* (2007) and modified for us, was used. In brief, increasing volumes of a c_Fe_ = 200 g L^−1^ solution of FeSO_4_·7H_2_O for Fe (II) were added to 15 ml of a potassium humate solution (28 g C org L^-1^). The pH was raised to 9.0 with KOH 1M solution. After one day in the dark, the pH was increased again to 9.0. After 2 h, the solutions were transferred to a 50-ml volumetric flask and the volume made up to 50 ml. The solutions were subsequently centrifuged at 10000 min^−1^ at room temperature for 10 min, and the supernatants filtered using 0.45 μm filters of cellulose acetate (Schleicher & Schuell). The complexed element was determined by inductively coupled plasma - optical emission spectrometry (ICP-OES) with a 5110 ICP-OES (Agilent Technologies, U.S.A.).


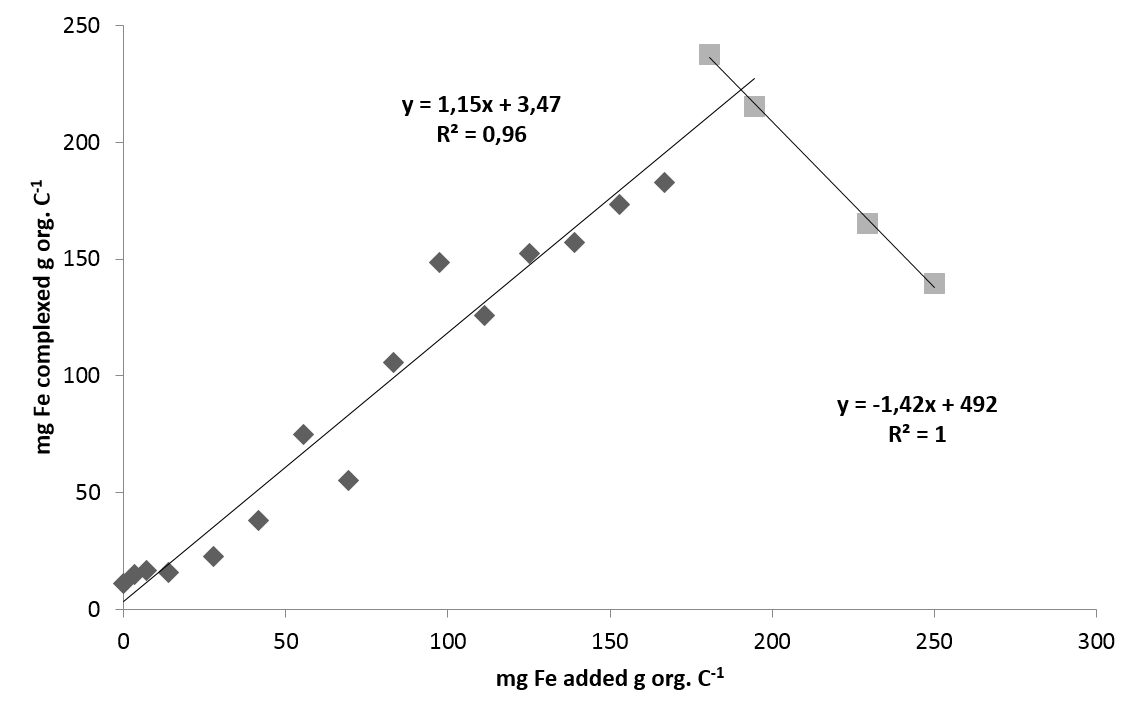


**Figure SM1**: Typical tritation curve for the determination of the maximum complexing capacity (Fe-MCC) of the potassium humate used with Fe (III).

**Reference**

Villén, M., Lucena, J.J., Cartagena, M.C., Bravo, R., García-Mina, J. and de la Hinojosa, M.I.M. (2007). Comparison of two analytical methods for the evaluation of the complexed metal in fertilizers and the complexing capacity of complexing agents. *J. Agric. Food Chem* 55, 5746–5753. doi.org/10.1021/jf070422t

**Determination of Fe using ICP AES spectroscopy**

An axial ICP-AES 5100 spectrometer with an SPS4 auto sampler (Agilent Technologies, USA) was used for ICP-AES measurements with a low flow axial quartz torch with 2.4 mm inner diameter injector tube, a double-pass glass cyclonic spray chamber, a glass pneumatic nebulizer (Agilent Technologies, USA), and a Trident Internal Standard Kit (Glass Expansion, Australia). A peristaltic pump used the white/white polyvinyl chloride pump tube for feeding and the blue/blue one for drain. A Sc (20 mg/L) internal standard solution was added online (orange/blue polyvinyl chloride pump tube) to increase the accuracy of measurements. Conditions of ICP–AES measurements are presented in Table 1. Results were collected and processed by ICP Expert software 2.0.5 (Agilent Technologies, USA).

**Table SM1.** The conditions of ICP–AES measurements

| **Conditions for all lines registrations** | |
| --- | --- |
| RF power ( kW) | 1.40 |
| Plasma flow (L/min) | 18.0 |
| Axial flow (L/min) | 1.50 |
| Nebulizer flow (L/min) | 0.95 |
| Replicate read time (s) | 20 |
| Instrument stabilization delay (s) | 25 |
| Replicates | 6 |
| **Sample introduction settings** | |
| Sample uptake delay (s) | 25 |
| Pump rate (rpm) | 12 |

Deionized water (18.2 MΩ × cm from a Milli-Q Academic system, Millipore, France) was used for the preparation of all the solutions and washing. An iron standard solution, 1000 mg/L (High Purity Standards) was used for calibration in the range 0.01–100 mg/L. An internal standard solution of Sc (20 mg/L) was prepared from Sc standard solution, 1000 mg/L (High Purity Standards).

**XRD characterization of ^57^Fe-NFs**

According to the XRD data, no iron-containing phases were observed in the obtained samples. Sample F contains niter (alpha-KNO_3_, #71-1558) as a major crystalline phase, sample M – K_3_Na(SO_4_)_2_ and Na_2_SO_4_ , sample S - arcanite (K_2_SO_4_).

Obtained XRD patterns and results of leBail refinement are demonstrated on


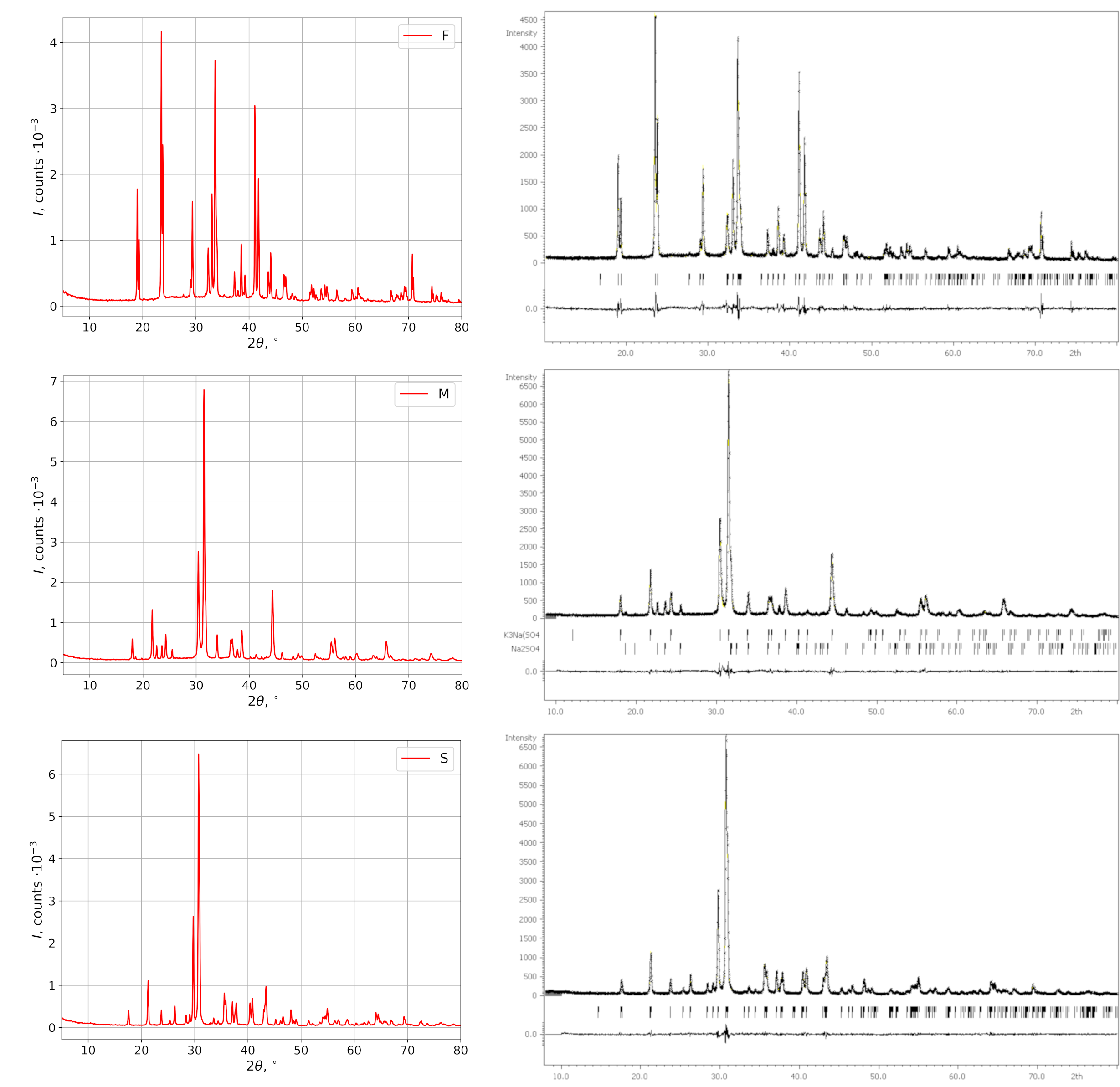
**Figure SM2.** XRD pattern of the product F compared to the KNO_3_ pattern (a), XRD pattern of the product M compared to K_3_Na(SO_4_)_2_ and Na_2_SO_4_ patterns (b), XRD pattern of the product S compared to the K_2_(SO_4_) pattern.

**XRD of the reference samples**

According to the XRD data, the reference sample of goethite contains pure goethite (*Pmcn*, a 5.4182, b 9.1694, c 6.4389). Samples of ferrihydrite (Fh), obtained with pH value of 7 and 8, demonstrate XRD patterns, usual for the 2-line ferrihydrite.


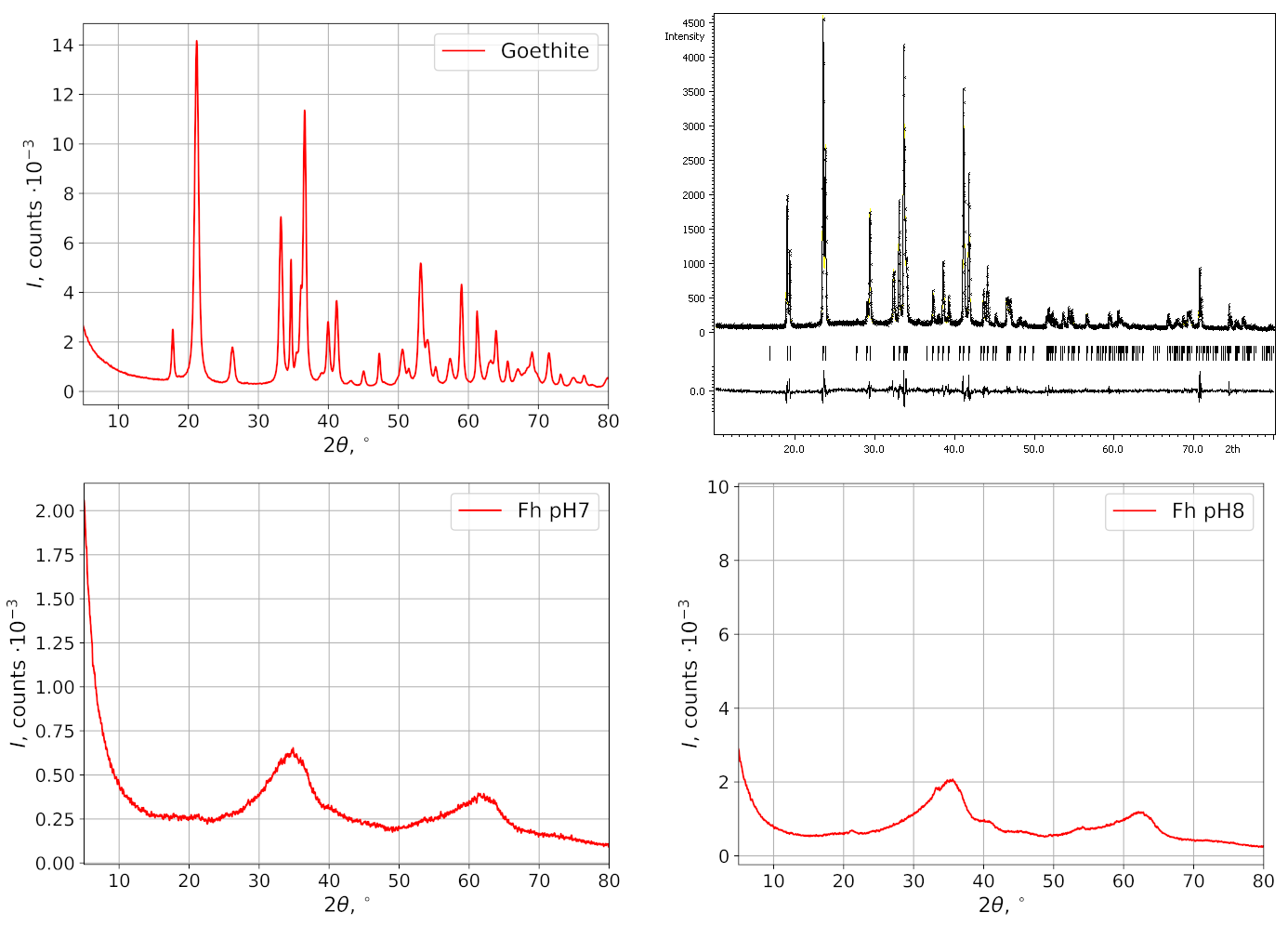


**Figure SM3.** XRD patterns of goethite and Fh preparations

**Electron Energy Loss Spectra**

The observed core loss lines of iron for the sample F are shown in Fig S4. For EFTEM imaging the M-line was chosen due to its high relative intensity.


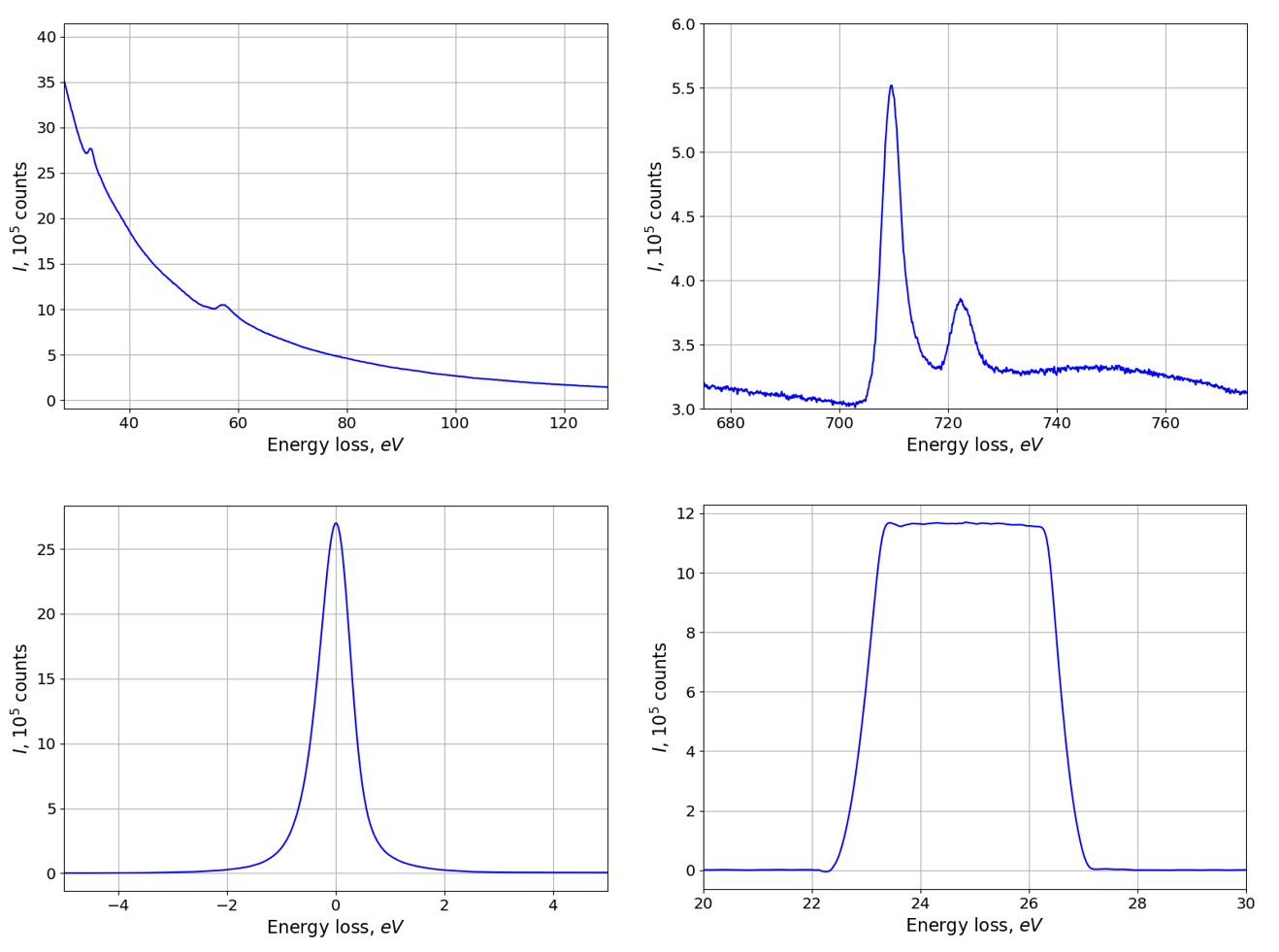


**Figure SM4**: EELS of the product F in different energy ranges, zero loss peak and the selected energy range (slit size) for the EFTEM.

**Zero-loss images**

In order to increase the contrast of iron-containing NP, the elastic (zero-loss) imaging was used. Obtained images for F and M samples are exemplified at Figure SM5.


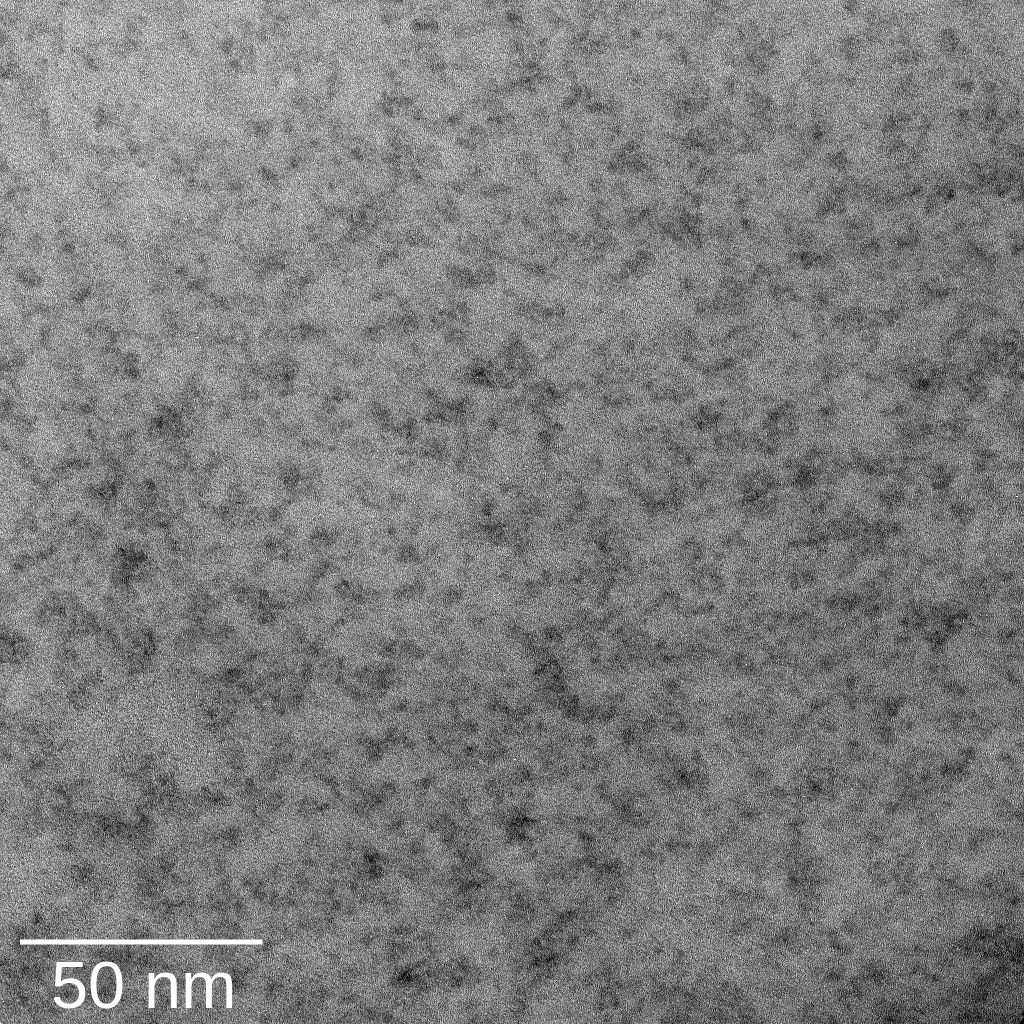

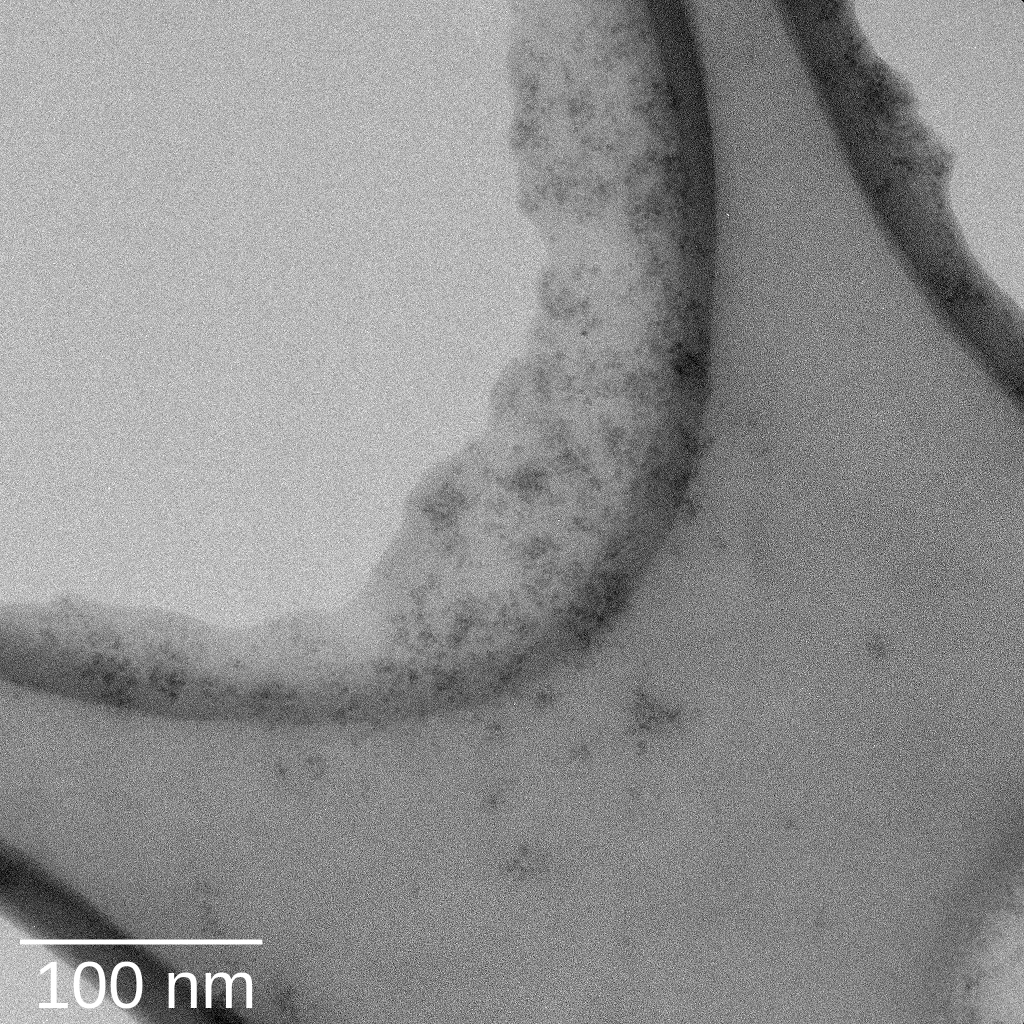


**Figure SM5.** Zero-loss EFTEM images of the F and M samples, respectively

**Electron diffraction data**

The selected areas and corresponding ED patterns are shown in Figure SM6.


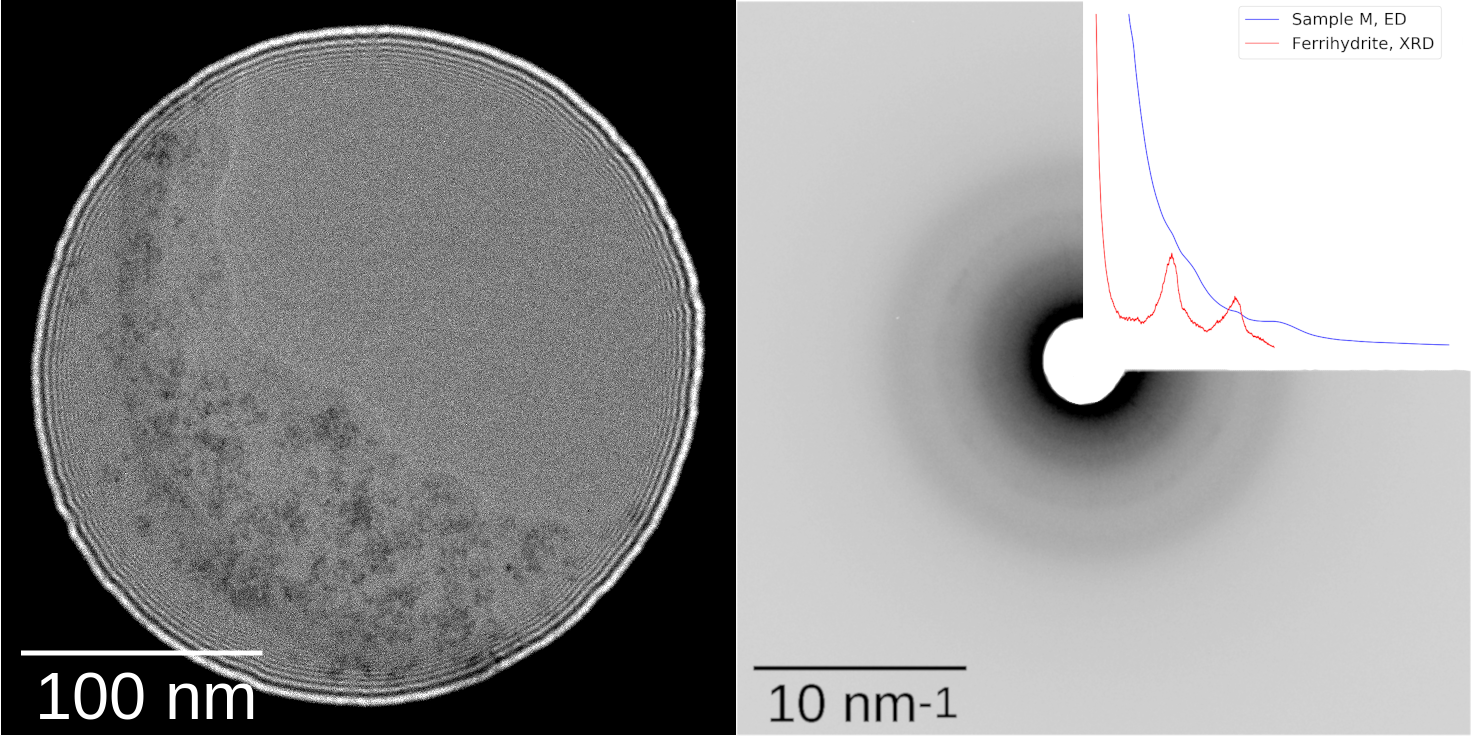

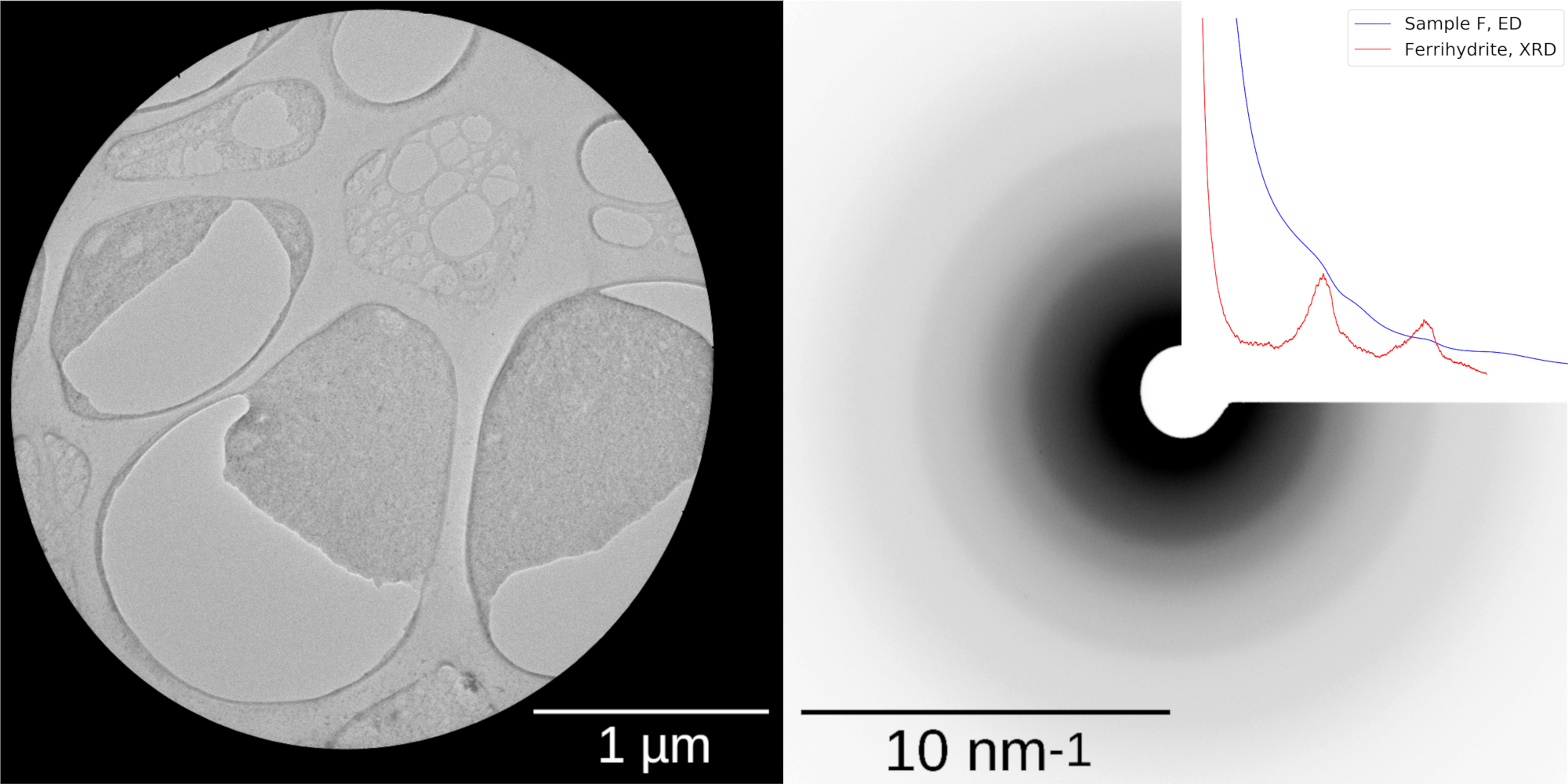


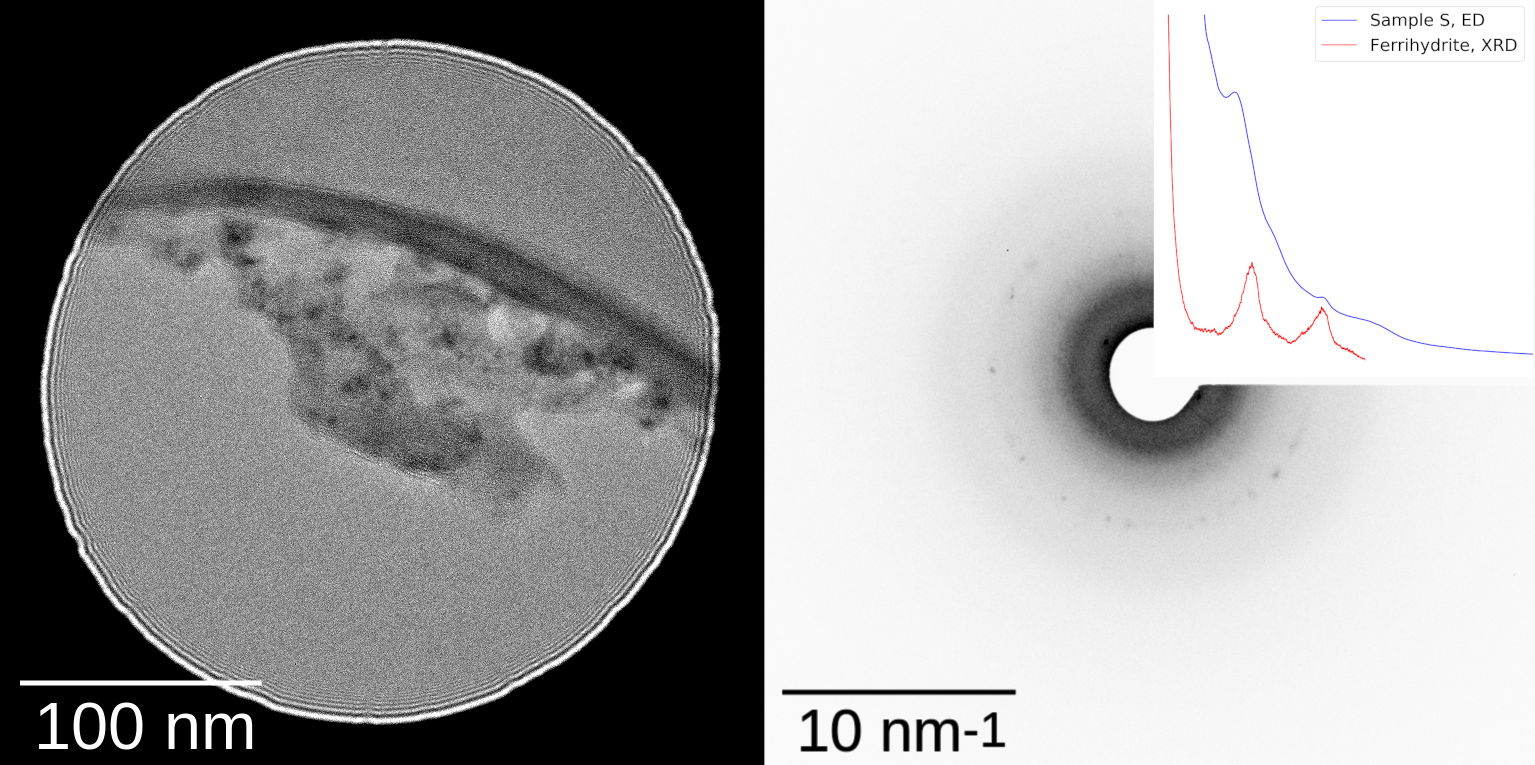


**Figure SM6.** SAED of F, M, and S samples: the selected area for the ED, and collected ED pattern with the integration result and XRD pattern of ferrihydrite inn the inset.

**XANES and EXAFS studies**

Fe K-line XANES spectra of F, M, S and reference samples are demonstrated in Figure SM9.

a
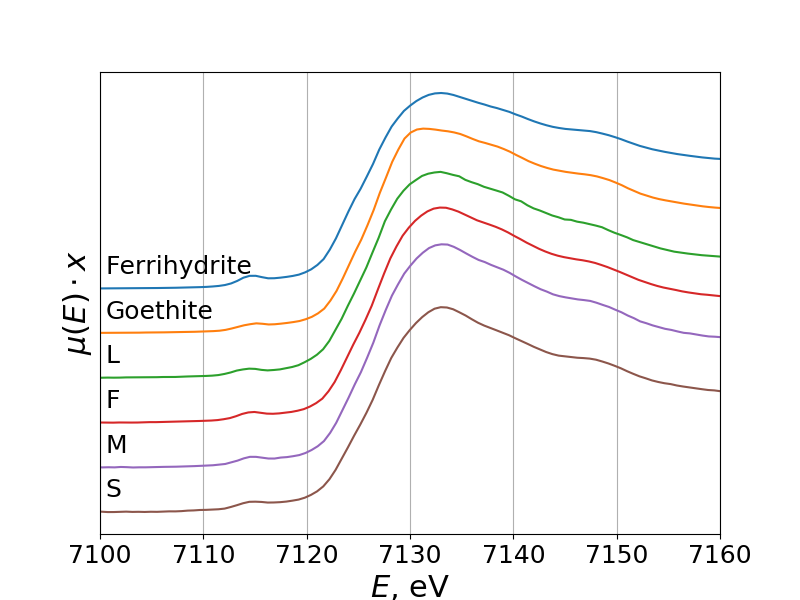
b
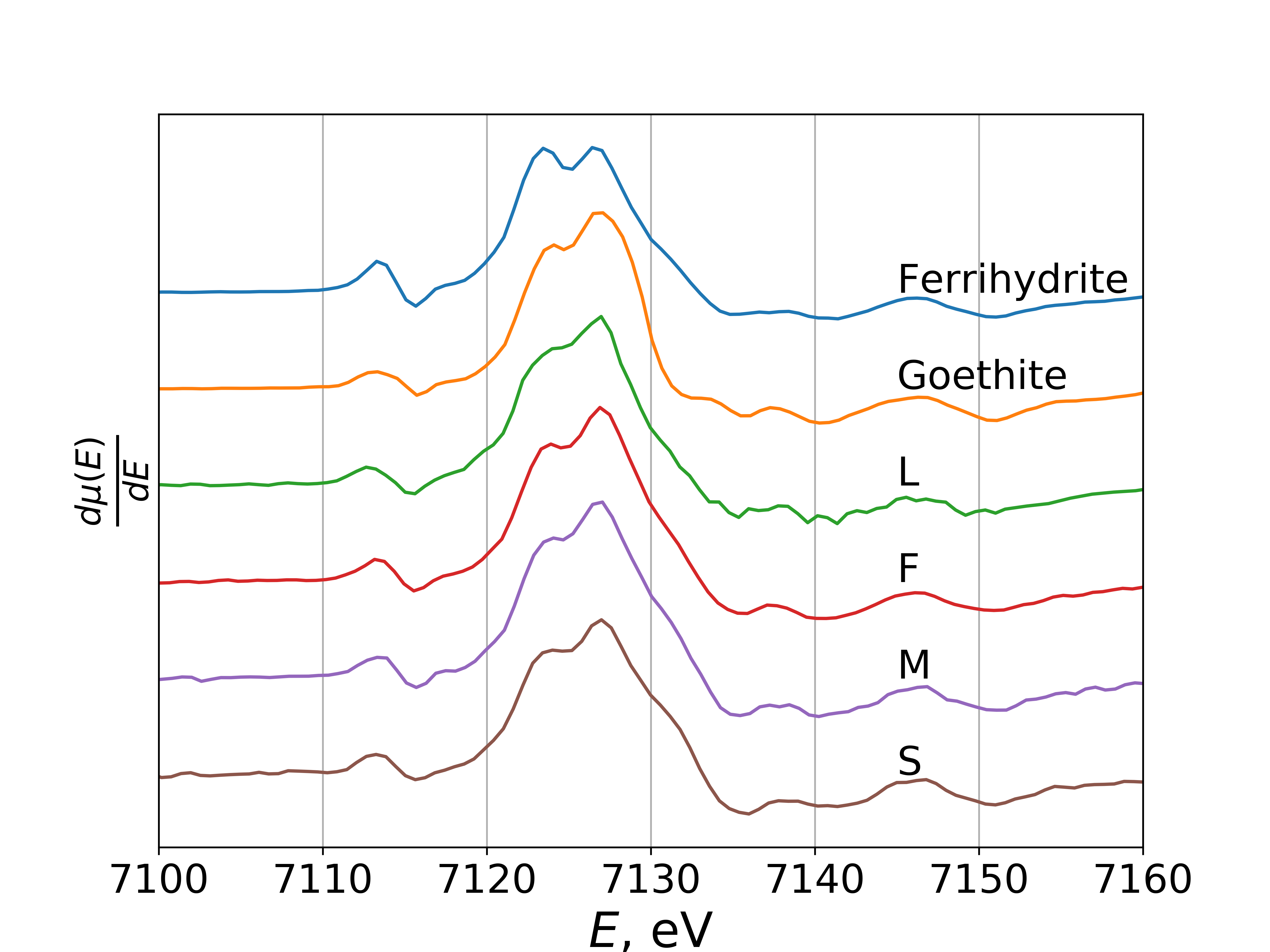


**Figure SM7.** Fe K-line XANES spectra (a) and the first derivatives of the XANES spectra (b) of three ^57^Fe-labelled nanofertilizers and reference samples of ferrihydrite, goethite, and the parent humate (L) (a)


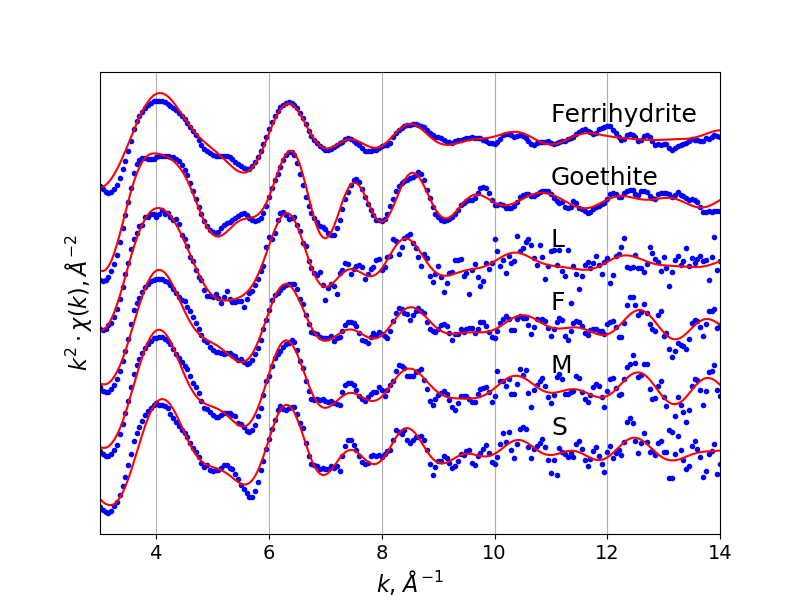
a
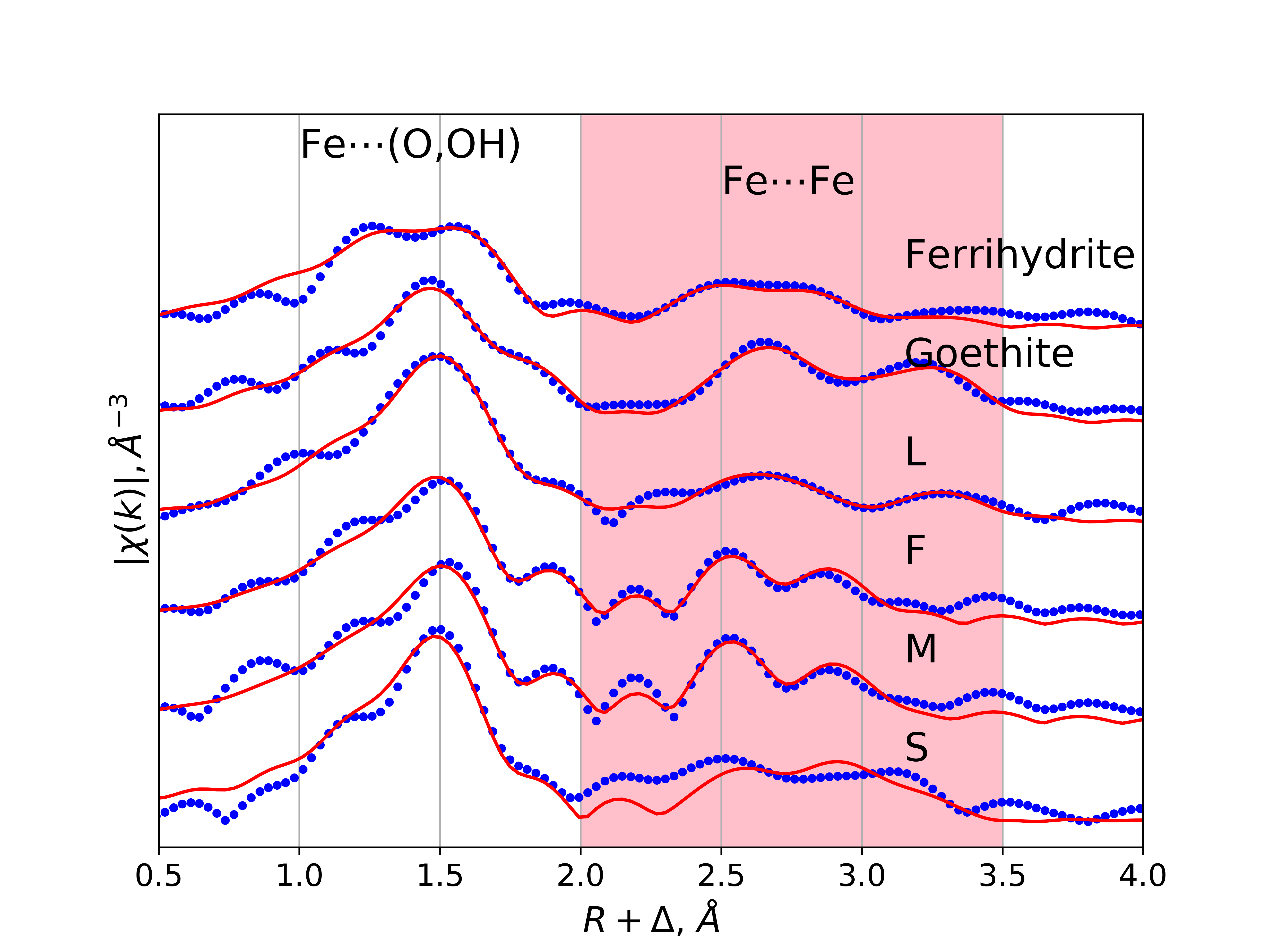
b

**Figure SM8.** EXAFS spectra in k- (a) and R-space (b) of three ^57^Fe-labelled nanofertilizers and reference samples of ferrihydrite, goethite, and the parent humate (L).

**Mössbauer spectroscopy characterization of reference samples (goethite and ferrihydrite) and ^57^Fe-NFs.**

The Mössbauer spectrum at room temperature for a reference goethite sample is a distorted sextet with asymmetrically broadened lines characteristic for microcrystalline of magnetically ordered substances with a high blocking temperature. The Mössbauer spectrum is satisfactorily described by the sextet by the many-state superparamagnetic relaxation model (Jones and Srivastava 1986) with parameters: δ=0.37 mm/s, Δ=-0.26 mm/s, H_hf_= 38.5 T (Figure SM9a). From the data obtained, taking the value of magnetic anisotropy energy constant equal to 10^3^ J/m^3^ (Shinjo 1966), one can estimate the size of goethite crystallites as 34.6 nm. The amount of the paramagnetic impurity phase was about 4%. At the boiling point of liquid nitrogen, the width of sextet lines is significantly reduced, which made it possible to describe the ferromagnetic part of the spectrum by a superposition of two sextets with hyperfine parameters corresponding to goethite (Mørup et al. 1983): δ_1_=0.47 mm/s, Δ_1_=-0.24 mm/s, H_hf1_= 49.6 T, S_1_=72%; δ_2_=0.48 mm/s, Δ_1_=-0.22 mm/s, H_hf1_= 48.0 T, S_1_=25%. The content of the paramagnetic phase of the impurity does not exceed 3% (Figure SM9b).

The Mössbauer spectrum of a control sample of ferrihydrite at room temperature is a symmetric paramagnetic doublet with markedly broadened lines. The description of the experimental spectrum using the quadrupole splitting distribution functions shows that the quadrupole splitting has a bimodal distribution (Figure SM10a). When the temperature drops to the boiling point of liquid nitrogen, due to the appearance of magnetic ordering in the structure of the ferrihydrite, the paramagnetic doublet increases noticeably, the distribution of quadrupole splitting turns into a unimodal representation with a simultaneous significant increase in the dispersion of the distribution. (FigureSM10b). In addition, at low temperature about 3% of a magnetically ordered phase is observed, with a magnetic hyperfine field H_hf_ = 48.2 T, corresponding to that observed in low-temperature spectra for ferrihydrite (Zhao et al. 1994). The results of the model description of the paramagnetic part of experimental spectra using a pair of nested symmetric doublets are presented in Table SM2.


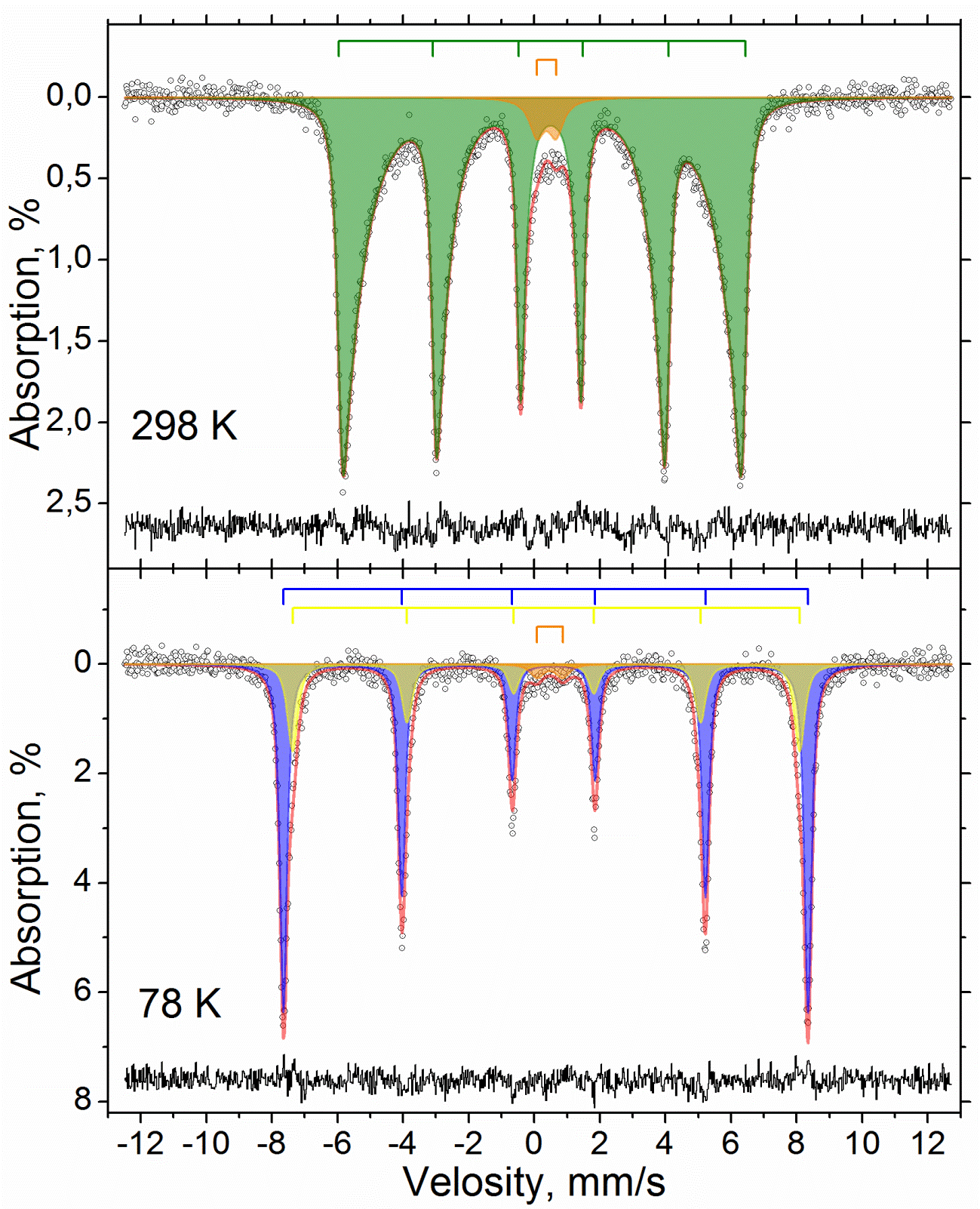


**Figure SM9.** Mössbauer spectra of the goethite sample recorded at 298 (a) and 78 (b) K.


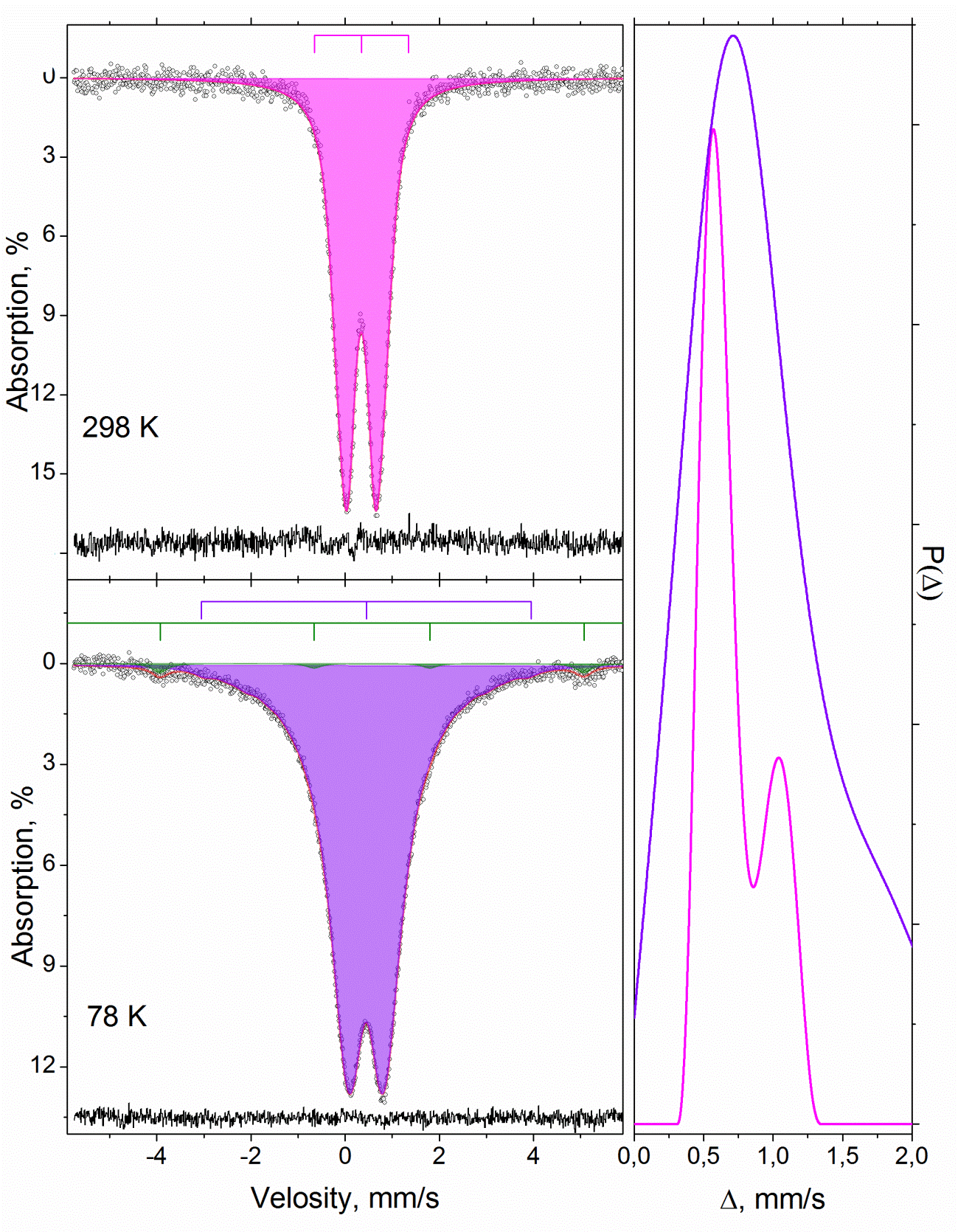


**Figure SM10.** Mössbauer spectra of the ferrihydrite sample recorded at 298 (a) and 78 (b) K and the quadrupole splitting distributions (c) for these spectra.


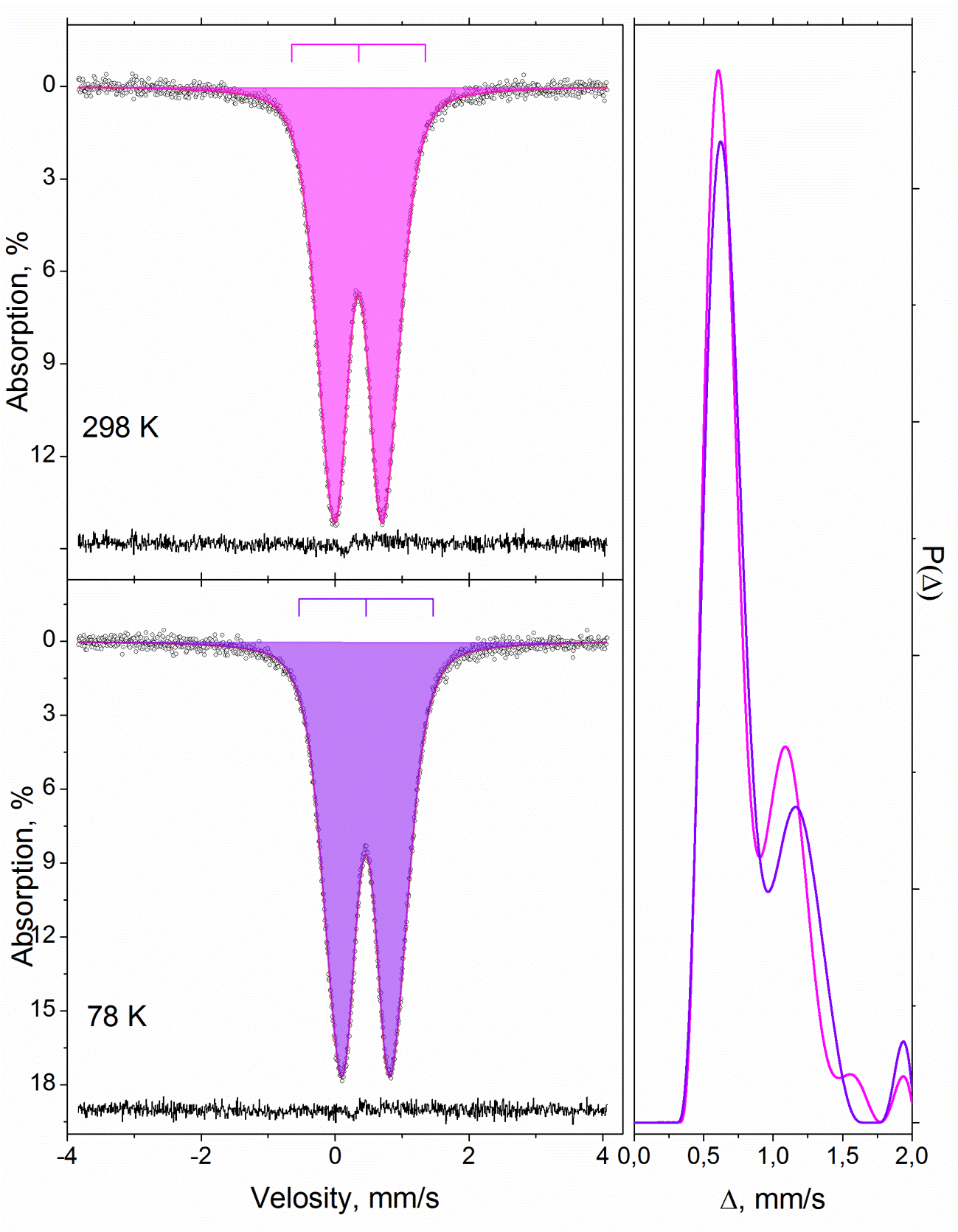


**Figure SM11.** Mössbauer spectra of the F sample recorded at 298 (a) and 78 (b) K and the quadrupole splitting distributions (c) for these spectra.

In addition to the non-model description of the paramagnetic part of the Mössbauer spectra for the three samples, a description of the model in the form of by superposition of two embedded symmetrical quadrupole doublets can be proposed (Table SM2).

**Table SM2**. Data of the Mössbauer spectra recorded at different temperatures

| Temperature, K | | 298 | | | | 78 | | | |
| --- | --- | --- | --- | --- | --- | --- | --- | --- | --- |
| Sample | Subspectrum | δ^*^ | Δ | Γ_exp_ | S | δ | Δ | Γ_exp_ | S |
|  |  | mm/s | | | % | mm/s | | | % |
| Ferrihydrite | 1 | 0.35±0.01 | 0.82±0.03 | 0.55±0.01 | 75±8 | 0.45±0.01 | 1.2±0.3 | 1.7±0.1 | 42±16 |
|  | 2 | 0.35±0.01 | 0.50±0.01 | 0.34±0.03 | 25±8 | 0.45±0.01 | 0.78±0.01 | 0.74±0.07 | 55±16 |
| M | 1 | 0.35±0.01 | 0.85±0.03 | 0.56±0.01 | 80±9 | 0.46±0.01 | 0.87±0.05 | 0.57±0.01 | 78±11 |
|  | 2 | 0.34±0.01 | 0.52±0.02 | 0.35±0.04 | 20±9 | 0.46±0.01 | 0.54±0.02 | 0.35±0.05 | 22±11 |
| F | 1 | 0.35±0.01 | 0.91±0.06 | 0.55±0.01 | 70±12 | 0.46±0.01 | 0.94±0.06 | 0.57±0.01 | 69±12 |
|  | 2 | 0.35±0.01 | 0.56±0.02 | 0.37±0.03 | 30±12 | 0.46±0.01 | 0.57±0.02 | 0.38±0.03 | 31±12 |
| S | 1 | 0.34±0.01 | 0.86±0.05 | 0.54±0.03 | 42±10 | 0.44±0.01 | 0.82±0.04 | 0.67±0.01 | 53±7 |
|  | 2 | 0.34±0.01 | 0.53±0.03 | 0.40±0.03 | 23±9 | 0.46±0.01 | 0.54±0.02 | 0.38±0.07 | 11±7 |
|  | 3 | 0.28±0.02 |  | 4.0±0.3 | 36±2 | 0.52±0.04 |  | 6.9±0.4 | 36±1 |

^*^δ, isomer shift; Δ, quadrupole splitting; Γ_exp_, line width; S, relative area of a subspectrum.

The hyperfine parameters of quadrupole doublets for all samples correspond to iron (III) atoms in the octahedral coordination environment of oxygen (Pankratov 2014). They are similar to one another, and are statistically poorly distinguishable (see Table SM2). Analyzing the hyperfine parameters of quadrupole doublets at two temperatures for all samples, we note a linear correlation of the width of the resonance lines of the quadrupole splitting (Figure SM12), which almost coincides with the previously observed for oxo-hydroxy compounds obtained in the presence of nanohydroxyapatite (Pankratov 2017).


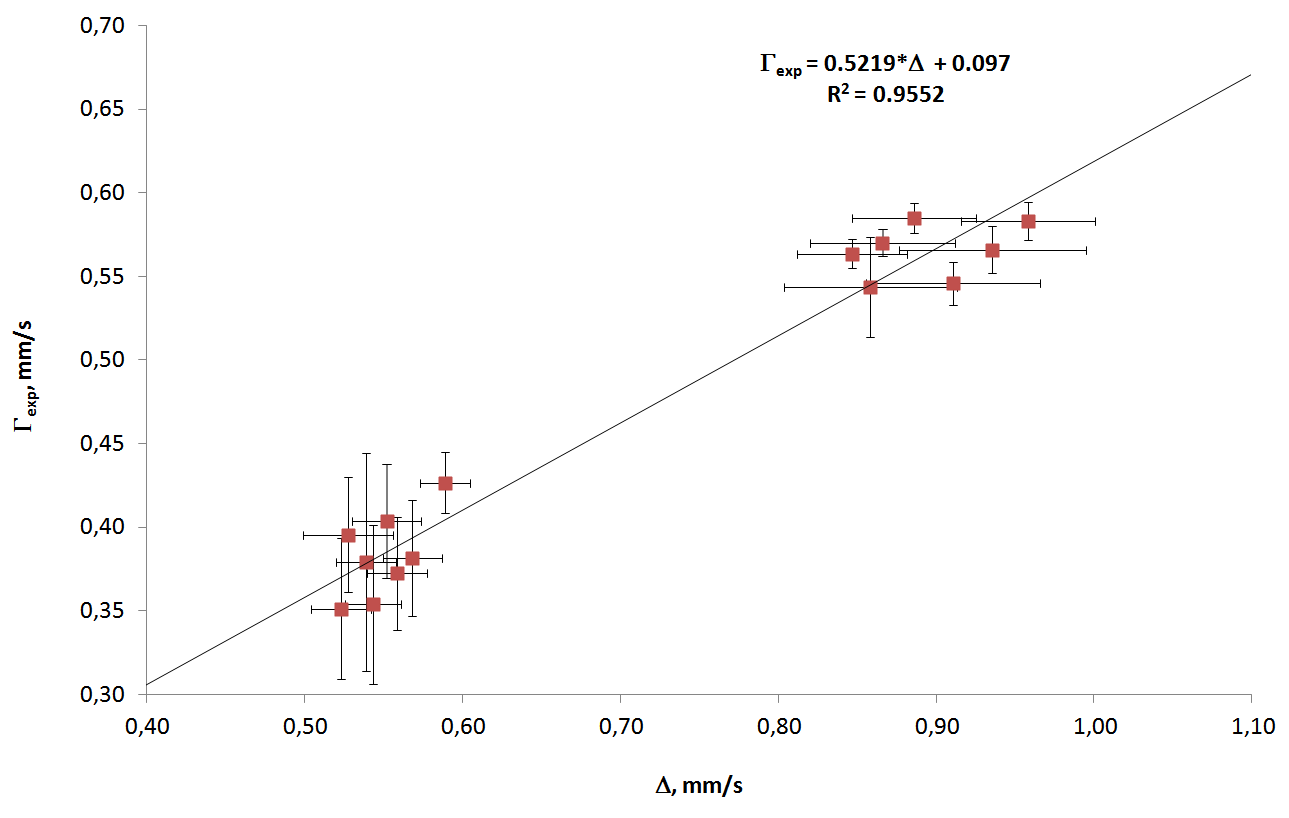


**Figure SM12.** Correlation of quadrupole splittings and widths of resonance lines for quadrupole doublets for Mössbauer spectra of the samples studied (data for different temperatures are combined, data for the first doublet of the S sample at 78 K were not taken into account).

In this paper, we came to the conclusion that the reason for this dependence is a partial contribution of the molecular-ion fragments of the series H_2_O-OH^-^-O^2-^ surrounding the iron atoms to the parameters of the Mössbauer spectra associated with their dynamic properties and electromagnetic interactions.

**References**

Jones, D.H. and Srivastava, K.K.P. (1986). Many-state relaxation model for the Mossbauer spectra of superparamagnets. *Phys. Rev. B: Condens. Matter Mater*. *Phys*. 34, 7542−7548. doi.org/10.1103/PhysRevB.34.7542

Mørup, S., Bo Madsen, M., Franck, J., Villadsen, J. and Koch, C.J.W. (1983). A new interpretation of Mössbauer spectra of microcrystalline goethite: "Super-ferromagnetism" or "super-spin-glass" behavior? *J. Magn. Magn. Mater.* 40, 163-174. doi.org/10.1016/0304-8853 (83)90024-0

Pankratov, D.A. (2014). Mössbauer study of oxo derivatives of iron in the Fe_2_O_3_-Na_2_O_2_ system. *Inorg. Mater*. 50, 82-89. doi.org/10.1134/S0020168514010154

Pankratov, D.A., Dolzhenko, V.D., Ovchenkov, E.A., Anuchina, M.M. and Severin, A.V. (2017). Properties of iron-containing nanohydroxyapatite-based composites. *Inorg. Mater*. 53, 89–98. doi.org/10.1134/S0020168517010125

Shinjo, T. (1996) Mössbauer effect in antiferromagnetic fine particles*. J. Phys. Soc. Jpn*. 21, 917-922 doi.org/10.1143/JPSJ.21.917

Zhao, J, Huggins, F.E., Feng, Z. and Huffman, G. (1994). Ferrihydrite: surface structure and its effects on phase transformation. *Clays. Clay. Miner*. 42, 737-746 doi.org/10.1346/CCMN.1994.0420610
